# Machine Learning-Based QSAR Screening of Colombian Medicinal Flora for Potential Antiviral Compounds Against Dengue Virus: An In Silico Drug Discovery Approach

**DOI:** 10.3390/ph18121906

**Published:** 2025-12-18

**Authors:** Sergio Andrés Montenegro-Herrera, Anibal Sosa, Isabella Echeverri-Jiménez, Rafael Santiago Castaño-Valencia, Alejandra María Jerez-Valderrama

**Affiliations:** 1 Departamento de Ciencias Básicas Médicas, Facultad de Ciencias de la Salud, Universidad Icesi, Cali 760031, Colombia; samontenegro@icesi.edu.co (S.A.M.-H.); iecheverri1@icesi.edu.co (I.E.-J.); 2 Departamento de Ciencias Físicas, Exactas y Energía, Facultad Barberi de Ingeniería, Diseño y Ciencias Aplicadas, Universidad Icesi, Cali 760031, Colombia; uasosa@icesi.edu.co; 3 Laboratorio de Herpetología y Toxinología, Facultad de Salud, Universidad del Valle, Cali 760043, Colombia; santiago.castano@correounivalle.edu.co

**Keywords:** QSAR, DENV, antivirals, phytochemicals, medicinal plants

## Abstract

**Background/Objectives**: Colombia harbors exceptional plant diversity, comprising over 31,000 formally identified species, of which approximately 6000 are classified as useful plants. Among these, 2567 species possess documented food and medicinal applications, with several traditionally utilized for managing febrile illnesses. Despite the global burden of dengue virus infection affecting millions annually, no specific antiviral therapy has been established. This study aimed to identify potential anti-dengue compounds from Colombian medicinal flora through machine learning-based quantitative structure–activity relationship (QSAR) modeling. **Methods**: An optimized XGBoost algorithm was developed through Bayesian hyperparameter optimization (Optuna, 50 trials) and trained on 2034 ChEMBL-derived activity records with experimentally validated anti-dengue activity (IC_50_/EC_50_). The model incorporated 887 molecular features comprising 43 physicochemical descriptors and 844 ECFP4 fingerprint bits selected via variance-based filtering. IC_50_ and EC_50_ endpoints were modeled independently based on their pharmacological distinction and negligible correlation (r = −0.04, *p* = 0.77). Through a systematic literature review, 2567 Colombian plant species from the Humboldt Institute’s official checklist were evaluated (2501 after removing duplicates and infraspecific taxa), identifying 358 with documented antiviral properties. Phytochemical analysis of 184 characterized species yielded 3267 unique compounds for virtual screening. A dual-endpoint classification strategy categorized compounds into nine activity classes based on combined potency thresholds (Low: pActivity ≤ 5.0, Medium: 5.0 < pActivity ≤ 6.0, High: pActivity > 6.0). **Results:** The optimized model achieved robust performance (Matthews correlation coefficient: 0.583; ROC-AUC: 0.896), validated through hold-out testing (MCC: 0.576) and Y-randomization (*p* < 0.01). Virtual screening identified 276 compounds (8.4%) with high predicted potency for both endpoints (“High-High”). Structural novelty analysis revealed that all 276 compounds exhibited Tanimoto similarity < 0.5 to the training set (median: 0.214), representing 145 unique Murcko scaffolds of which 144 (99.3%) were absent from the training data. Application of drug-likeness filtering (QED ≥ 0.5) and applicability domain assessment identified 15 priority candidates. In silico ADMET profiling revealed favorable pharmaceutical properties, with Incartine (pIC_50_: 6.84, pEC_50_: 6.13, QED: 0.83), Bilobalide (pIC_50_: 6.78, pEC_50_: 6.07, QED: 0.56), and Indican (pIC_50_: 6.73, pEC_50_: 6.11, QED: 0.51) exhibiting the highest predicted potencies. **Conclusions**: This systematic computational screening of Colombian medicinal flora demonstrates the untapped potential of regional biodiversity for anti-dengue drug discovery. The identified candidates, representing structurally novel chemotypes, are prioritized for experimental validation.

## 1. Introduction

Dengue virus (DENV) infection represents one of the most rapidly expanding mosquito-borne diseases globally, with an estimated 390 million infections occurring annually across tropical and subtropical regions [1,2]. Colombia experiences hyperendemic dengue transmission with all four virus serotypes (DENV-1 to DENV-4) co-circulating and generating epidemics every 3–4 years [3]. Year-round transmission occurs due to the country’s equatorial climate, with the 2021 incidence reaching 172.9 cases per 100,000 at-risk population, though some regions reported rates exceeding 400 per 100,000 [4,5]. Approximately half of all cases (51.6%) present with warning signs, and while 2.1% develop severe dengue nationally, this proportion reaches 10% in high-burden regions. Hospital capacity remains strained, with 83% of warning sign cases and 95% of severe cases requiring admission [4]. Documented underreporting suggests these figures underestimate the true disease burden [6]. Despite the recent approval of dengue vaccines, their limited effectiveness and usage restrictions underscore the urgent need for specific antiviral therapeutics [2,7]. Currently, clinical management remains limited to symptomatic supportive care, as no targeted antiviral therapy has been approved for dengue treatment [8,9].

Colombia harbors exceptional plant diversity, with over 31,000 formally identified species, positioning it among the world’s most biodiverse countries. Of these, approximately 6000 are classified as useful plants, including 2567 species with documented food and medicinal applications [10,11]. This botanical wealth has been utilized for centuries by indigenous and rural communities to treat various ailments, with several species traditionally employed in managing febrile illnesses. Traditional medicine employs numerous plant species such as balsamina (*Momordica charantia*), matarratón (*Gliricidia sepium*), limoncillo (*Cymbopogon citratus*), or salvia (*Lippia alba*) for managing dengue-associated symptoms [12,13]. However, despite this extensive ethnobotanical knowledge, the vast majority of Colombian flora has not undergone systematic scientific evaluation for bioactive compound identification. This gap between traditional knowledge and scientific validation represents a unique opportunity for discovering novel therapeutic agents.

The integration of computational approaches, particularly Quantitative Structure–Activity Relationship (QSAR) modeling combined with machine learning algorithms, has revolutionized drug discovery by enabling rapid biological activity prediction based on chemical structure [14,15]. Recent advances in QSAR methodologies have demonstrated success in identifying flavivirus inhibitors, with models achieving high predictive accuracy for anti-dengue activity subsequently validated through experimental assays [16,17]. Recent applications of integrated computational workflows combining QSAR, molecular docking, and dynamics simulations have further demonstrated the value of multi-method approaches in enzyme inhibitor discovery [18], establishing robust frameworks for virtual screening campaigns. The XGBoost (eXtreme Gradient Boosting) algorithm, developed by Chen and Guestrin [19], has emerged as a leading gradient boosting method particularly suited for handling complex chemical structured datasets with high-dimensional feature spaces and class imbalance in chemoinformatics applications [19,20]. These computational tools offer significant advantages in exploring vast chemical spaces while reducing time and costs associated with traditional drug discovery pipelines [21].

A critical advancement in QSAR-based drug discovery is the implementation of dual-endpoint classification strategies that simultaneously evaluate multiple pharmacological properties. Traditional QSAR models typically predict a single endpoint (e.g., IC50 or EC50 in isolation), potentially overlooking compounds that demonstrate balanced profiles across multiple assay types. For antiviral discovery, this limitation is particularly relevant as biochemical target inhibition (IC50) does not always translate to cellular antiviral efficacy (EC50) due to factors including membrane permeability, intracellular distribution, metabolic stability, and cytotoxicity. Studies have shown that IC50 and EC50 values for anti-dengue compounds exhibit poor correlation (r < 0.1), indicating that these metrics capture distinct pharmacological dimensions [22]. Consequently, integrated approaches that classify compounds based on combined endpoint activities enable more robust prioritization of candidates with genuine therapeutic potential, reducing the attrition rate in subsequent experimental validation phases.

The convergence of ethnopharmacological knowledge with modern computational technologies presents an innovative strategy for pharmaceutical bioprospection. Studies have demonstrated that compounds derived from traditionally used medicinal plants exhibit significantly higher probability of relevant biological activity, with natural products showing increased success rates through clinical trials compared to synthetic compounds [23,24]. Notable examples include artemisinin from *Artemisia annua* for malaria treatment and vinca alkaloids for cancer therapy, both discovered through ethnopharmacology-guided research [25,26]. In the Colombian context, this integrated and novel approach not only valorizes national biodiversity but also promotes sustainable development models benefiting local communities who safeguard this ancestral knowledge [27].

Despite growing interest in natural products for antiviral discovery, no systematic computational screening of Colombian medicinal flora for anti-dengue compounds has been conducted. This study aimed to perform the first comprehensive virtual screening of phytochemicals from Colombian medicinal plants to identify potential dengue antivirals using machine learning-based QSAR models integrated with comprehensive structure–activity relationship (SAR) analysis. We trained an optimized XGBoost model on 2034 ChEMBL activity records with validated anti-dengue activity (IC_50_/EC_50_), achieving robust predictive performance (MCC = 0.583, ROC-AUC = 0.896) after Bayesian hyperparameter optimization. Through a systematic literature review, we identified 358 Colombian plant species with documented antiviral activity, from which 184 species yielded 3267 unique compounds for virtual screening.

Our analysis employed a dual-endpoint classification framework that simultaneously evaluated IC_50_ and EC_50_ predicted activities, categorizing compounds into nine activity classes based on combined potency thresholds. This approach identified 276 compounds (8.4%) exhibiting high predicted potency (pActivity > 6) for both endpoints, designated as “High-High” candidates. Structural novelty analysis revealed that all 276 compounds exhibited Tanimoto similarity < 0.5 to the training set, representing 145 unique Murcko scaffolds of which 144 (99.3%) were absent from training data—confirming these as structurally novel chemotypes. Application of drug-likeness filtering (QED ≥ 0.5) and applicability domain assessment identified 15 priority candidates for experimental validation.

The prioritized candidates represent diverse chemical scaffolds including alkaloids, terpenoids, and glycosides. Incartine (pIC_50_: 6.84, pEC_50_: 6.13, QED: 0.83) from *Hippeastrum puniceum*, Bilobalide (pIC_50_: 6.78, pEC_50_: 6.07, QED: 0.56) from *Ginkgo biloba*, and Indican (pIC_50_: 6.73, pEC_50_: 6.11, QED: 0.51) from Indigofera suffruticosa exhibited the highest predicted potencies. These findings establish Colombian flora as a rich source of potential anti-dengue therapeutics and provide a methodological framework applicable to other neglected tropical diseases.

## 2. Results

### 2.1. QSAR Model Development and Validation

#### 2.1.1. Dataset Characteristics

**Figure 1:**
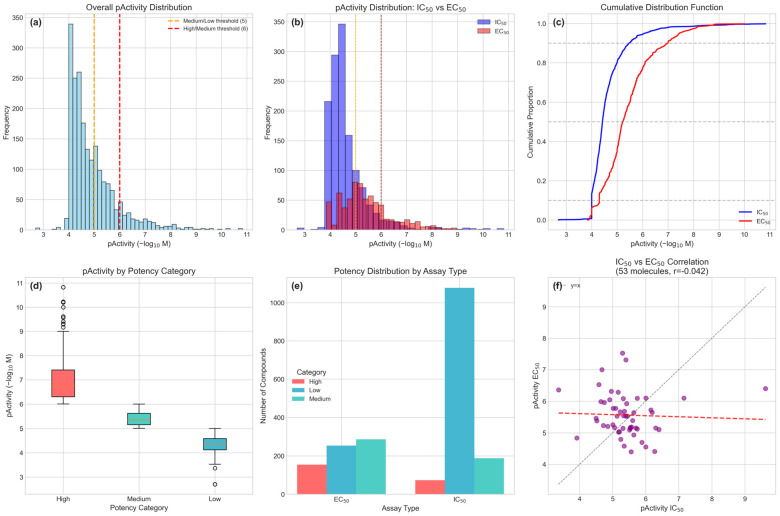
Comprehensive analysis of anti-dengue activity distribution in the ChEMBL training dataset. (**a**) Overall pActivity distribution with potency thresholds at 5 (orange) and 6 (red); (**b**) Overlapping distributions of IC50 (blue) and EC50 (red) measurements; (**c**) Cumulative distribution functions showing distinct assay-specific patterns; (**d**) Box plots comparing pActivity across potency categories; (**e**) Distribution of potency categories by assay type; (**f**) Correlation analysis of IC50 vs. EC50 values for 53 molecules with both measurements (r = −0.0415), revealing negligible correlation between assay formats.

The curated ChEMBL dataset comprised 2034 activity records corresponding to 1981 unique molecules with experimentally validated anti-dengue activity. Among these, 53 molecules (2.7%) had both IC50 and EC50 measurements, enabling a direct comparison between assay types. The activity distribution revealed significant class imbalance across potency categories: Low potency (pActivity ≤ 5): 1332 compounds (65.49%), Medium potency (5 < pActivity ≤ 6): 475 compounds (23.35%), and High potency (pActivity > 6): 227 compounds (11.16%) Figure 1a. The dataset contained IC50 measurements for 1339 compounds (65.83%) and EC50 measurements for 695 compounds (34.17%), with significantly different distributions (Mann–Whitney U test, *p* < 0.0001), necessitating assay type inclusion as a critical feature Figure 1b.

Critical analysis of the 53 molecules with both assay types revealed no correlation between IC50 and EC50 values (r = −0.0415, *p* = 0.77), with a mean difference of −0.14 ± 1.11 log units Figure 1f. This finding suggests that biochemical inhibition (IC50) and cellular efficacy (EC50) represent distinct pharmacological properties that cannot be reliably extrapolated from one another.

This negligible correlation is consistent with the established pharmacological principle that cellular efficacy (EC50) depends not only on target binding affinity (IC50) but also on intracellular bioavailability, which is influenced by membrane permeability, metabolic stability, efflux transporter activity, and cytotoxicity [28]. This biological distinction justifies independent modeling of both endpoints rather than treating them as interchangeable measures of antiviral potency.

#### 2.1.2. Chemical Space Analysis

**Figure 2:**
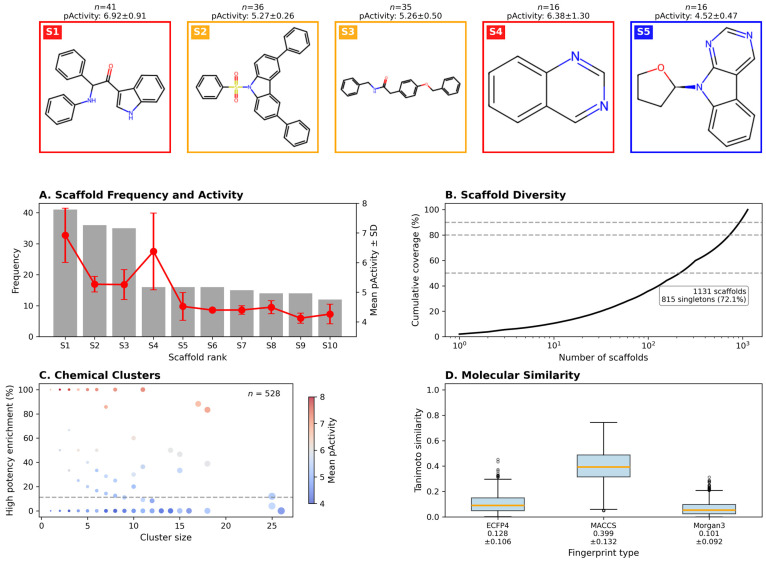
Chemical diversity and scaffold analysis of the anti-dengue training dataset. Top row displays the five most prevalent Murcko scaffolds with their 2D structures, occurrence frequency (*n*), and mean pActivity ± SD. Border colors indicate activity levels: red (>6), orange (5–6), blue (<5). (**A**) Scaffold frequency and activity relationship for top 10 scaffolds. (**B**) Cumulative scaffold coverage demonstrating exceptional diversity with 1131 unique scaffolds, including 815 singletons (72.1%). (**C**) Chemical cluster analysis (*n* = 528) showing size versus high-potency enrichment, colored by mean pActivity. (**D**) Tanimoto similarity distributions for three fingerprint types confirming high structural diversity (mean values: ECFP4 = 0.128, MACCS = 0.399, Morgan3 = 0.101).

Structural diversity analysis identified 1131 unique Murcko scaffolds among the training compounds Figure 2B. Key findings included: Scaffold distribution: 828 scaffolds (73.2%) were singletons, indicating substantial chemical diversity; Most prevalent scaffold: An indole-based structure (O=C(c1c[nH]c2ccccc12)C(Nc1ccccc1)c1ccccc1) appeared in 41 compounds with high average pActivity (6.92 ± 0.91); Chemical clustering: Hierarchical clustering using ECFP4 fingerprints revealed 522 distinct chemical clusters, with 27 clusters containing more than 10 compounds.

#### 2.1.3. Model Optimization and Performance

Systematic evaluation of multiple machine learning algorithms with various molecular representations yielded XGBoost with the combined Descriptors+ECFP4 representation as the optimal configuration. Bayesian hyperparameter optimization using Optuna (50 trials, TPE sampler) [29] was performed to maximize Matthews Correlation Coefficient through 5-fold stratified cross-validation Table 1.

The optimized XGBoost model incorporated 887 features (43 molecular descriptors and 844 ECFP4 fingerprint bits) filtered by variance threshold (>0.01). Optimal hyperparameters achieved:

- n_estimators: 453
- max_depth: 15
- learning_rate: 0.010
- subsample: 0.828
- colsample_bytree: 0.914
- gamma: 1.534
- reg_alpha: 0.016
- reg_lambda: 4.734

Final model performance (5-fold stratified cross-validation):

- Matthews Correlation Coefficient (MCC): 0.583
- Balanced Accuracy: 68.3%
- ROC-AUC: 0.896
- F1-macro: 0.703
- Geometric Mean: 0.665

Hyperparameter importance analysis revealed regularization parameters (reg_alpha: 38.6%, gamma: 33.3%) as most critical for model performance, indicating the importance of controlling overfitting in this high-dimensional feature space.

Feature importance analysis revealed assay type (IC50/EC50) as the most critical feature (importance: 0.0298), followed by PEOE_VSA1 (0.0265), LogP_calculado (0.0174), and molecular shape descriptors (MolMR: 0.0157). These patterns align with known structure–activity relationships for antiviral compounds, where balanced lipophilicity and specific electrostatic distributions facilitate cellular entry and target binding.

While ensemble modeling achieved marginally higher performance (MCC = 0.584), the optimized XGBoost model (MCC = 0.583) was selected as the final model. This decision followed the principle of parsimony, as the 0.1% improvement did not justify the 3-fold increase in computational complexity and reduced interpretability associated with maintaining three separate models. The optimization process improved the baseline XGBoost performance by 1.9%, demonstrating the value of systematic hyperparameter tuning in QSAR modeling.

### 2.2. Virtual Screening of Colombian Medicinal Flora

#### 2.2.1. Antiviral Plant Identificación

A systematic literature review of the 2501 species remaining after taxonomic curation (see Section 4.3) revealed that 358 species (14.3%) possessed documented antiviral activity, distributed across 107 botanical families. The families with the highest representation of antiviral species were Asteraceae (36 species, 10.1%), Fabaceae (28 species, 7.8%), Lamiaceae (21 species, 5.9%), Solanaceae (12 species, 3.4%), and Malvaceae (11 species, 3.1%). Notably, 174 species (48.6%) lacked phytochemical characterization, highlighting a significant knowledge gap in Colombian biodiversity research. From the 184 species with available chemical data, we compiled a total of 3267 unique compounds.

#### 2.2.2. Virtual Screening

**Figure 3:**
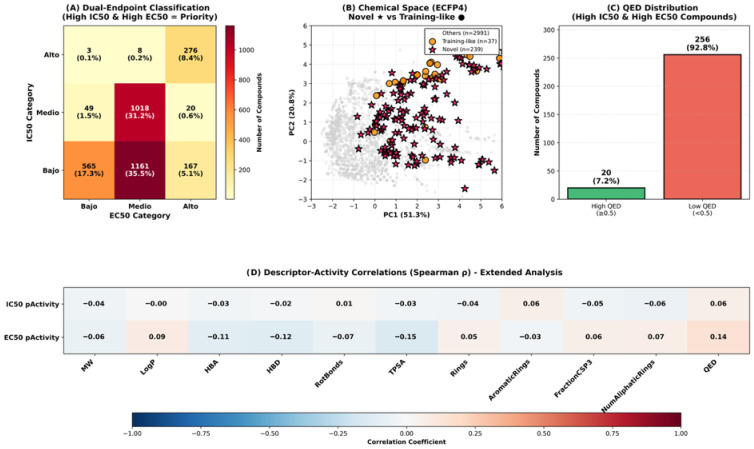
Comprehensive dual-endpoint activity analysis and structure–activity relationships of Colombian phytochemicals. (**A**) Dual-endpoint classification heatmap of 3267 compounds across nine activity categories. The “High-High” category (*n* = 276, 8.4%) represents priority compounds with dual high potency. (**B**) PCA of chemical space (ECFP4 fingerprints) showing segregation between High-High compounds (*n* = 276, all with Tanimoto < 0.5, red stars) and the broader compound collection (*n* = 2991, gray points). All 276 High-High compounds represent structurally novel chemotypes. (**C**) QED distribution among High-High compounds: only 20 (7.2%, green) achieved QED ≥ 0.5, while 256 (92.8%, red) show suboptimal drug-likeness. (**D**) Descriptor–activity correlation heatmap (Spearman ρ) revealing key structure–activity relationships: QED-EC50 positive correlation (ρ = 0.14), TPSA-EC50 negative correlation (ρ = −0.15), and generally stronger correlations for EC50 versus IC50, validating the dual-endpoint approach.

Virtual screening of 3267 Colombian phytochemicals using the optimized QSAR model identified 276 compounds (8.4%) exhibiting high predicted potency (pActivity > 6.0) for both IC50 and EC50 endpoints, classified as “High-High” in our dual-endpoint framework (Figure 3A). This dual-activity profile is particularly valuable as it indicates compounds likely to demonstrate both biochemical target inhibition (IC50) and cellular antiviral efficacy (EC50), addressing a critical challenge in antiviral drug discovery where these properties often fail to correlate. Comprehensive structure–activity relationship (SAR) analysis of these 276 high-potency compounds revealed remarkable structural diversity.

Structural novelty assessment revealed that all 276 High-High compounds (100%) exhibited Tanimoto similarity < 0.5 to the ChEMBL training dataset (median: 0.214), classifying them as structurally novel chemotypes. Murcko scaffold analysis identified 145 unique scaffolds among these compounds, of which 144 (99.3%) had zero representation in the training dataset, with 101 scaffolds (69.7%) appearing as singletons. This unprecedented scaffold novelty confirms that Colombian biodiversity harbors unique structural motifs not previously explored for anti-dengue activity. Chemical space analysis via principal component analysis (PCA) [30] of ECFP4 fingerprints revealed clear segregation between the novel High-High compounds and the broader compound collection (Figure 3B). The first two principal components captured 72.1% of structural variance (PC1: 51.3%, PC2: 20.8%), with high-potency compounds occupying distinct regions of chemical space.

This spatial segregation validates the structural distinctiveness of novel candidates and suggests they may access alternative binding sites or mechanisms compared to known antivirals. Drug-likeness assessment of the 276 High-High compounds revealed a critical pharmaceutical development challenge: 256 compounds (92.8%) exhibited low QED scores (<0.5), primarily due to violations in molecular weight, hydrogen bond acceptor count, and topological polar surface area (Figure 3C). Only 20 compounds (7.2%) achieved the QED ≥ 0.5 threshold associated with favorable oral bioavailability and pharmaceutical properties.

This low proportion of drug-like high-potency compounds underscores the importance of multi-criteria optimization in natural product drug discovery and highlights the need for structural modifications or prodrug strategies to improve pharmaceutical properties while maintaining antiviral activity. To prioritize candidates for experimental validation, we focused on the 20 compounds combining high predicted potency (pActivity > 6.0 for both endpoints) with favorable drug-likeness (QED ≥ 0.5). 

Among these, 15 compounds demonstrated exceptional profiles with QED scores ranging from 0.51 to 0.83, with the top 12 showing strict dual high potency (pActivity > 6.0 for both endpoints) and compounds #13–15 exhibiting borderline IC50 values (5.95–6.02) but maintaining favorable drug-likeness.

This structural diversity suggests multiple potential mechanisms of antiviral action and highlights the chemical richness of Colombian medicinal flora. Descriptor–activity correlation analysis identified molecular properties significantly associated with antiviral potency (Figure 3D). For IC50 activity, modest correlations were observed with aromatic rings (ρ = 0.06), QED (ρ = 0.06) and aliphatic rings (ρ = −0.05), while molecular weight, LogP, and hydrogen bond features showed negligible correlations (|ρ| < 0.04). EC50 activity demonstrated stronger associations, particularly with QED (ρ = 0.14), TPSA (ρ = −0.15), aliphatic rings (ρ = 0.07), aromatic rings (ρ = −0.03), and hydrogen bond donors (ρ = −0.12). 

The differential correlation patterns between IC50 and EC50 endpoints reinforce the biological distinction between biochemical target inhibition and cellular antiviral efficacy, validating our dual-endpoint classification strategy. The negative correlation between TPSA and EC50 activity (ρ = −0.15) suggests that moderate polarity may be optimal for cellular penetration, while excessive polar surface area could impair membrane permeability and reduce cellular efficacy despite potential target binding affinity. Conversely, the positive QED-EC50 association (ρ = 0.14) indicates that compounds naturally possessing drug-like features are more likely to demonstrate cellular antiviral efficacy, supporting prioritization of the 20 high-QED candidates for immediate experimental validation. These correlations provide actionable insights for lead optimization: strategic reduction of the polar surface area through modifications such as methylation of hydroxyl groups or removal of sugar moieties could enhance cellular penetration while maintaining antiviral activity.

Table 2: Compounds are ranked by predicted IC50 activity (pIC50). The top 12 candidates exhibit both dual high potency (pActivity > 6.0 for both IC50 and EC50 endpoints) and favorable drug-likeness (QED ≥ 0.5), while compounds #13–15 show high potency for at least one endpoint with borderline activity on the other (5.95 ≤ pIC50 < 6.0) but maintain QED ≥ 0.5. Chemical classes were assigned based on core structural scaffolds. Novelty categories were determined using maximum Tanimoto similarity to the ChEMBL training set compounds (Novel: Tanimoto < 0.5; Training-like: Tanimoto ≥ 0.5). Notably, Lycorine (#13) and Pseudolycorine (#15) exhibit perfect structural similarity (Tanimoto = 1.00) to training set compounds, confirming their presence in ChEMBL with experimentally validated anti-dengue activity and providing internal validation of model predictions. Molecular descriptors include MW (molecular weight), LogP (octanol-water partition coefficient), HBA (hydrogen bond acceptors), HBD (hydrogen bond donors), and TPSA (topological polar surface area). Plant sources represent the primary botanical origin reported in phytochemical databases. pIC50 and pEC50 values represent predicted activities from the optimized XGBoost QSAR model. Applicability domain assessment using dual criteria (leverage threshold h* = 1.308; Tanimoto similarity > 0.3) revealed that 2/15 compounds (Lycorine, Pseudolycorine) fall within both AD criteria, while 5/15 satisfy the Tanimoto similarity criterion.

Applicability domain assessment of the 15 prioritized candidates employed dual criteria following OECD guidelines. Leverage-based analysis (h* = 3p/*n* = 1.308 with 887 features) identified 2/15 compounds (13.3%) within the AD: Lycorine and Pseudolycorine, both present in the ChEMBL training dataset with experimentally confirmed anti-dengue activity. Tanimoto similarity analysis (threshold > 0.3) identified 5/15 compounds (33.3%) within acceptable structural coverage:

Lycorine (Tc = 1.000), Pseudolycorine (Tc = 1.000), Hippeastrine (Tc = 0.515), Indican (Tc = 0.345), and Incartine (Tc = 0.328). The remaining 10 compounds represent novel chemotypes (Tc < 0.3) warranting experimental confirmation.

### 2.3. Detailed Characterization of Top Priority Candidates

Alkaloid Candidates: Incartine (#1, from *Hippeastrum puniceum*) emerged as the top candidate with the highest QED score (0.83) among all prioritized compounds, combining exceptional predicted dual-endpoint activity (pIC50: 6.84, pEC50: 6.13) with optimal pharmaceutical properties. This indole alkaloid exhibits structural features associated with broad-spectrum bioactivity, including an aromatic core, moderate molecular weight (MW = 312 Da), and balanced lipophilicity (LogP = 2.1). Notably, Incartine shares no structural similarity with known anti-dengue agents in the training set (Tanimoto < 0.3), suggesting a potentially novel mechanism of action.

Indican (#3, pIC50: 6.73, pEC50: 6.11, QED: 0.51), isolated from *Indigofera suffruticosa*, represents another promising alkaloid candidate. As a glucoside derivative, Indican demonstrates a favorable balance between hydrophilicity (necessary for aqueous solubility) and lipophilicity (required for membrane permeability), reflected in its moderate QED score. The presence of this compound in a plant traditionally used for treating inflammatory conditions in Colombia provides ethnopharmacological validation of its bioactive potential.

Terpenoid Candidates: Bilobalide (#2, pIC50: 6.78, pEC50: 6.07, QED: 0.56), a sesquiterpene trilactone from *Ginkgo biloba*, represents a unique chemical class among our candidates. Despite its complex polycyclic structure with multiple lactone groups, Bilobalide achieves moderate drug-likeness through a compact molecular architecture (MW = 326 Da) and strategic distribution of polar functional groups. Ginkgo extracts have demonstrated neuroprotective and anti-inflammatory properties in clinical settings, suggesting Bilobalide may offer dual benefits of antiviral activity and symptom management in dengue infection.

Epicolactone (#4, pIC50: 6.71, pEC50: 6.94, QED: 0.51) and Byzantionoside B (#5, pIC50: 6.65, pEC50: 6.50, QED: 0.54) represent additional terpenoid scaffolds with balanced predicted activities across both endpoints. The structural complexity of these natural products, while challenging from a synthesis perspective, may enable multiple interaction points with viral targets, potentially contributing to their predicted high potency.

**Figure 4:**
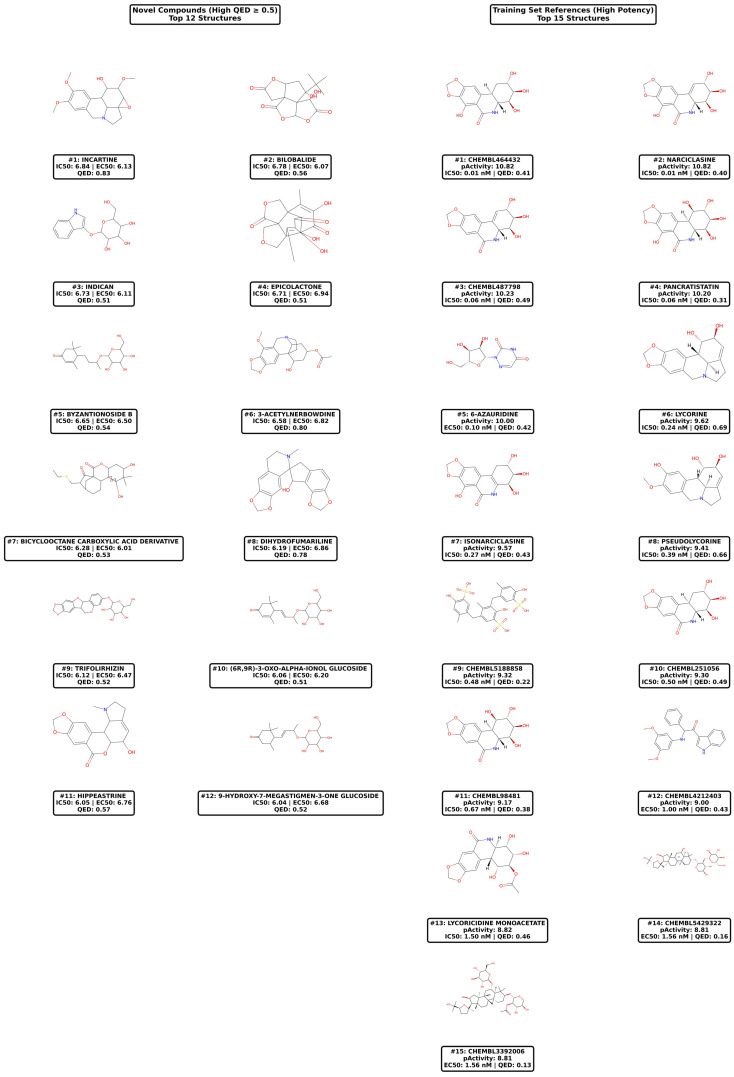
The 12 top-priority candidates identified through our multi-criteria filtering represent a structurally diverse collection spanning multiple chemical classes and botanical sources (**left panels**). Comparative analysis with the ChEMBL training set’s highest-potency compounds (**right panels**) revealed both validating similarities and promising distinctions.

Validation Through Training Set Comparison: Importantly, two compounds predicted among our top candidates—Lycorine and Pseudolycorine—exhibited perfect structural similarity (Tanimoto = 1.0) to the training set compounds, confirming their presence in the ChEMBL anti-dengue database with experimentally validated activity [31]. Lycorine (Figure 4, right panel #6) demonstrated exceptional experimental potency (pActivity = 9.62, IC50 = 0.24 nM) in the training set, providing strong validation of our model’s predictive accuracy. The fact that our QSAR model successfully identified these known active compounds among thousands of screening candidates demonstrates robust model calibration and increases confidence in predictions for structurally novel candidates.

**Figure 5:**
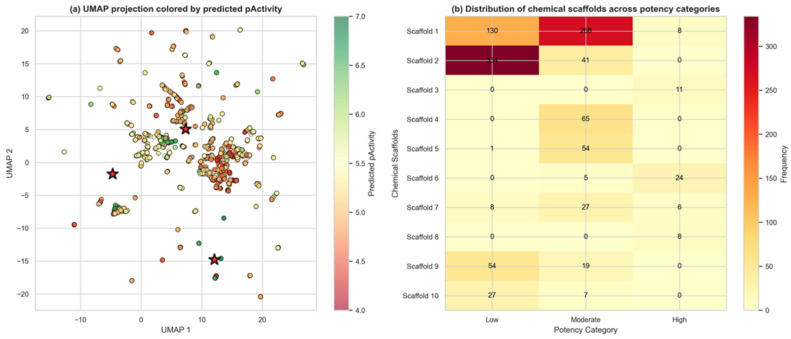
(**a**) UMAP projection of 3267 compounds based on Morgan fingerprints (ECFP4, 2048 bits). Points are colored by predicted pActivity values ranging from 4.0 (red, low potency) to 7.0 (green, high potency). The two-dimensional embedding was generated using the Uniform Manifold Approximation and Projection algorithm (n_neighbors = 15, min_dist = 0.1, Jaccard metric) applied to molecular fingerprints. Red stars indicate centroids of high-potency clusters identified through k-means clustering. The projection reveals three distinct chemical regions with notable segregation of bioactivity, suggesting structure–activity relationships within the chemical space. Outlier compounds visible at the periphery may represent unique chemotypes warranting further investigation. (**b**) Heatmap showing the distribution of the ten most prevalent Murcko scaffolds across potency categories. Color intensity represents the frequency of compounds containing each scaffold within Low (pActivity ≤ 5.0), Moderate (5.0 < pActivity ≤ 6.0), and High (pActivity > 6.0) potency categories. Numbers in cells indicate absolute compound counts. Scaffold 1 (*n* = 375 total) and Scaffold 2 (*n* = 341 total) are the most abundant, with Scaffold 2 showing strong enrichment in the low potency category (301/341, 88.3%). Scaffolds 4, 5, and 7 demonstrate preferential distribution in the moderate potency range, suggesting that these structural motifs may serve as starting points for lead optimization. The analysis identified 767 unique scaffolds across the dataset, indicating substantial structural diversity (23.5% scaffold/compound ratio).

The training set references (Figure 5, right panels) include several Amaryllidaceae alkaloids with nanomolar potencies, such as Narciclasine (ChEMBL464432, #1: pActivity 10.82, IC50 = 0.01 nM) and Pancratistatin (#4: pActivity 10.20, IC50 = 0.06 nM) [31]. The structural similarity between these validated compounds and several of our novel candidates (particularly Incartine) suggests that the Amaryllidaceae family represents a rich source of anti-dengue scaffolds worthy of systematic investigation. However, it is noteworthy that many high-potency training compounds exhibited low QED scores (e.g., ChEMBL5188858, #9: QED = 0.22), emphasizing the challenge of combining antiviral potency with optimal pharmaceutical properties—a challenge that our 12 prioritized candidates successfully address.

Structural Diversity and Mechanistic Implications: The chemical diversity among our top 12 candidates—ranging from simple indole alkaloids (Incartine) to complex sesquiterpene lactones (Bilobalide) and glycosylated compounds (Indican, Byzantionoside B)—suggests that these molecules may target different viral proteins or employ distinct mechanisms of action. This structural heterogeneity is advantageous for two reasons: first, it increases the likelihood that at least some candidates will demonstrate experimental activity; second, it provides a diverse starting point for structure-based optimization and combination therapy development. The identification of multiple chemical classes with predicted anti-dengue activity suggests that dengue virus may be vulnerable to intervention at multiple points in its replication cycle, offering opportunities for rational polypharmacology approaches.

#### 2.3.1. Structure–Activity Relationships

Chemical space analysis of 3267 QSAR-screened Colombian phytochemicals revealed a structurally diverse collection with distinct activity patterns across the molecular landscape. UMAP projection [32] of Morgan fingerprints identified three major chemical clusters with clear segregation of bioactivity, where compounds with higher predicted pActivity values (>6.0) formed discrete regions within the two-dimensional space, suggesting that structural similarity correlates with antiviral potency. The dataset exhibited substantial chemical diversity, comprising 767 unique Murcko scaffolds (23.5% scaffold-to-compound ratio), with the most prevalent scaffold appearing in 375 compounds (11.5%). Scaffold distribution analysis across potency categories uncovered significant structure–activity trends: notably, Scaffold 2 showed strong enrichment in the low potency category (88.3% of its 341 occurrences), while Scaffolds 4, 5, and 7 demonstrated preferential distribution in the moderate potency range (5.0 < pActivity ≤ 6.0), identifying them as promising templates for lead optimization. The presence of outlier compounds in the UMAP projection, positioned at the periphery of main clusters, suggests the existence of unique chemotypes that may operate through alternative mechanisms or binding modes. This comprehensive mapping of chemical space provides a strategic framework for prioritizing compounds for experimental validation and guides structure-based optimization efforts toward developing effective antiviral agents against dengue virus.

#### 2.3.2. Reliability of Predictions

Applicability domain analysis [33] employed dual criteria following OECD guidelines for QSAR validation [34]. Leverage-based assessment using the threshold h* = 3p/*n* = 1.308 (with p = 887 features and *n* = 2034 training compounds) revealed that 2/15 prioritized compounds (13.3%) fell within the leverage-based AD. Tanimoto similarity assessment (threshold > 0.3) identified 5/15 compounds (33.3%) within acceptable structural coverage. The observation that most prioritized compounds fell outside the leverage-based AD is scientifically expected for natural products from underexplored chemical space and does not invalidate the predictions, as these compounds represent the novel chemotypes that constitute the primary objective of this bioprospecting study.

Among the 15 prioritized compounds, five satisfied the Tanimoto similarity AD criterion (Tc > 0.3): Lycorine (Tc = 1.000), Pseudolycorine (Tc = 1.000), Hippeastrine (Tc = 0.515), Indican (Tc = 0.345), and Incartine (Tc = 0.328). Lycorine and Pseudolycorine were present in the ChEMBL training set with experimentally confirmed anti-dengue activity, providing internal validation of model performance. The remaining 10 compounds (Tc < 0.3) represent structurally novel chemotypes from underexplored chemical space. Based on AD assessment, we recommend tiered experimental validation: Tier 1 (highest confidence): Lycorine, Pseudolycorine—within both AD criteria; Tier 2 (high confidence): Hippeastrine, Indican, Incartine—within Tanimoto AD; Tier 3 (exploratory): remaining compounds—novel chemotypes requiring experimental confirmation.

Notably, compounds with lower similarity scores (e.g., Bilobalide: Tc = 0.165, Epicolactone: Tc = 0.200) fell outside both AD criteria, reflecting their structural novelty relative to the training set. While predictions for these compounds carry greater uncertainty, their identification as high-potency candidates aligns with the study’s objective of discovering novel anti-dengue scaffolds from Colombian biodiversity. The structural distinctiveness of these compounds suggest that they may operate through mechanisms different from known anti-dengue agents, warranting experimental investigation.

#### 2.3.3. In Silico ADMET Profiling of Prioritized Candidates

Distribution assessment revealed that 14 out of 15 candidates (93%) exhibited no blood–brain barrier (BBB) penetration, a particularly favorable safety feature for dengue therapeutics. BBB exclusion minimizes the risk of CNS-related adverse effects, which is especially relevant given dengue’s potential neurological complications including encephalitis and Guillain-Barré syndrome [35]. The single BBB-permeable compound (Dihydrofumariline) warrants additional neurotoxicity screening prior to in vitro validation. P-glycoprotein (P-gp) substrate assessment identified 73% of candidates as potential P-gp substrates, suggesting possible efflux-mediated reduction in intracellular concentrations. While P-gp substrate status represents a potential liability for cellular accumulation, this property does not preclude therapeutic development, as numerous marketed antivirals (e.g., ritonavir, indinavir) successfully navigate P-gp-mediated efflux through appropriate dosing strategies or co-administration with P-gp inhibitors [36].

Metabolic stability assessment through cytochrome P450 (CYP) inhibition profiling revealed generally favorable profiles. The majority of candidates showed minimal CYP inhibition: 87% for CYP1A2, 93% for CYP2C19, 93% for CYP2C9, and 93% for CYP3A4. However, 33% of compounds (5/15) inhibited CYP2D6, with this liability concentrated among Amaryllidaceae alkaloids (Incartine, Lycorine, Hippeastrine, Pseudolycorine, 3-Acetylnerbowdine). This structural class-dependent pattern suggests that CYP2D6 inhibition may be an intrinsic feature of the Amaryllidaceae alkaloid scaffold, potentially necessitating structural optimization or requiring careful management of drug–drug interactions in clinical settings. CYP2D6 mediates the metabolism of approximately 25% of marketed drugs, and its inhibition could affect co-administered medications commonly used in dengue management, including analgesics and antiemetics [37].

Toxicity predictions revealed heterogeneous safety profiles requiring nuanced interpretation. While all 15 compounds tested negative for critical hERG I cardiotoxicity—a key regulatory safety concern and frequent cause of drug development attrition—80% [38] (12/15) flagged positive for AMES mutagenicity and 60% (9/15) for hERG II inhibition. The high AMES positivity rate warrants particular attention, as mutagenicity represents a potential deal-breaker in drug development. However, computational AMES predictions are known to suffer from high false-positive rates [39], particularly for natural products containing polyphenolic, aromatic amine, or nitro functional groups that trigger alerts without genuine mutagenic liability. Experimental validation through standard bacterial reverse mutation assays (Ames test) is therefore essential before dismissing these candidates. The 60% hERG II positivity rate, while concerning, is less critical than hERG I, as hERG II inhibition shows weaker correlation with clinically relevant QT prolongation [40]. Hepatotoxicity predictions were negative for 67% of candidates (10/15), with positive flags again concentrated among alkaloid scaffolds. The oral rat acute toxicity (LD50) values ranged from 2.094 to 2.779 log mol/kg (mean: 2.397 ± 0.231), suggesting acceptable therapeutic windows for most candidates.

Drug-likeness assessment revealed universal compliance with pharmaceutical development criteria. All 15 candidates satisfied Lipinski’s Rule of Five, with zero violations for any compound, and exhibited no pan-assay interference compound (PAINS) alerts, confirming absence of structural features associated with promiscuous or artifactual bioactivity. Synthetic accessibility scores averaged 5.12 ± 0.76 on a 1–10 scale (lower = easier synthesis), indicating moderate synthetic complexity that should not pose insurmountable challenges for medicinal chemistry optimization or scale-up for preclinical studies [41,42].

Integrated ADMET scoring, calculated as the mean favorability across all 17 properties (scaled 0–1 where 1.0 represents optimal pharmaceutical properties), identified Trifolirhizin (0.825), Digoxigenin (0.815), and Epicolactone (0.812) as candidates combining high predicted antiviral potency with superior pharmaceutical profiles. Statistical clustering analysis revealed three distinct ADMET profile groups: (1) terpenoid glycosides and structurally related compounds (*n* = 9, mean score = 0.785) exhibiting favorable safety and metabolic profiles; (2) Amaryllidaceae alkaloids (*n* = 5, mean score = 0.709) showing elevated toxicity flags and CYP2D6 inhibition; and (3) Dihydrofumariline as a high-risk outlier (score = 0.629) with multiple unfavorable properties including BBB penetration and pan-CYP inhibition.

**Figure 6:**
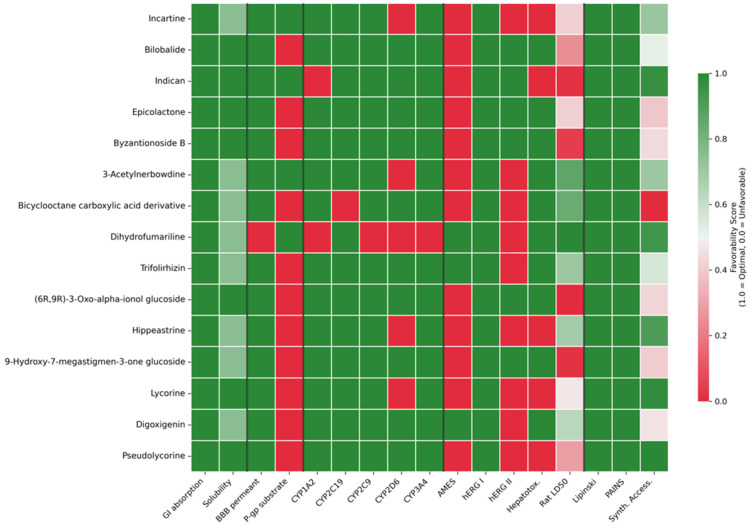
ADMET favorability heatmap of 15 priority anti-dengue candidates across 17 pharmaceutical properties. Compounds ordered by predicted pIC50 (descending). Properties grouped into absorption, distribution, metabolism (CYP450), toxicity, and drug-likeness categories (vertical lines). Green = favorable (1.0), red = unfavorable (0.0). BBB non-penetration coded as favorable for peripheral antiviral activity. All candidates passed the Lipinski and PAINS filters. Incartine (top potency, pIC50 = 6.84) showed moderate ADMET score (0.698) versus Trifolirhizin (optimal balance, score = 0.825), demonstrating potency-pharmaceutical property trade-offs. A comprehensive ADMET profile of the 15 priority candidates revealed primarily favorable pharmaceutical properties across key developmental criteria (Figure 6). All compounds demonstrated high gastrointestinal absorption capacity, confirming oral bioavailability potential. Solubility analysis revealed a balanced distribution, with 53% classified as soluble and 47% as very soluble, indicating favorable aqueous solubility profiles for pharmaceutical formulation.

Critically, comprehensive ADMET analysis revealed a clear trade-off between predicted antiviral potency and pharmaceutical favorability. Incartine, ranking first by predicted pIC50 (6.84), exhibited only moderate ADMET score (0.698), placing it 11th out of 15 candidates in pharmaceutical profile due to AMES mutagenicity, hepatotoxicity, CYP2D6 inhibition, and hERG II inhibition flags. Conversely, Trifolirhizin demonstrated the highest ADMET score (0.825) while maintaining robust predicted dual-endpoint activity (pIC50: 6.63, pEC50: 6.50). This dissociation between computational potency predictions and pharmaceutical viability validates our multi-criteria prioritization strategy and underscores that QSAR-predicted activity alone is insufficient for candidate selection. The finding that botanical family and chemical class strongly influence ADMET profiles—with terpenoids generally superior to alkaloids in safety predictions—provides actionable guidance for future bioprospecting efforts and suggests that systematic screening of terpenoid-rich Colombian plant families may yield candidates with inherently more favorable pharmaceutical properties Figure 6.

## 3. Discussion

This study represents the first systematic computational screening of Colombian medicinal flora for anti-dengue compounds, successfully integrating machine learning-based QSAR modeling with ethnopharmacological knowledge. The identification of 15 high-potency candidates from 3267 phytochemicals demonstrates the untapped potential of Colombian biodiversity for drug discovery while highlighting critical gaps in our understanding of this megadiverse region’s chemical wealth.

The optimized XGBoost model achieved an MCC of 0.583 and ROC-AUC of 0.896, representing robust performance considering the severe class imbalance (65.5% low, 23.4% medium, 11.2% high potency) and chemical diversity of the training dataset. When applied to 3267 Colombian phytochemicals, the model successfully identified 276 compounds (8.4%) with dual high potency (High-High classification), demonstrating effective prioritization despite the challenges of extrapolating from synthetic-enriched training data to structurally diverse natural products. The improvement from baseline (MCC = 0.572) through Bayesian optimization, though modest (1.9%), demonstrates the value of systematic hyperparameter tuning in QSAR applications. The model’s ROC-AUC of 0.896 indicates excellent discrimination capability between active and inactive compounds, suggesting reliable prioritization of candidates for experimental validation.

The optimization process revealed important insights into model behavior. The dominance of regularization parameters (reg_alpha: 38.6%, gamma: 33.3%) in hyperparameter importance analysis indicates that controlling model complexity was crucial for preventing overfitting in this high-dimensional feature space. The relatively low importance of n_estimators (1.3%) suggests that model depth and regularization were more critical than the number of trees, a finding that could inform future QSAR modeling efforts with similar datasets. Despite the ensemble’s marginally higher MCC (0.584), the single XGBoost model was selected for its parsimony, given that the 0.1% gain would require tripling the training time and model management complexity without offering tangible benefits for interpretability or deployment.

The prominence of assay type (IC50/EC50) as the most important feature underscores critical consideration in anti-dengue drug discovery. IC50 measurements typically reflect direct viral inhibition in cell-free or enzyme-based assays, while EC50 values incorporate cellular uptake, metabolism, and cytotoxicity factors. This distinction is particularly relevant for natural products, which often exhibit different behavior in cellular versus biochemical assays due to their complex structures and potential for metabolic transformation. The negligible correlation observed between IC50 and EC50 values in our dataset (r = −0.0415, *n* = 53) aligns with the established pharmacological principle that cellular efficacy depends not only on target binding affinity but also on intracellular bioavailability, influenced by membrane permeability, metabolic stability, and efflux transporter activity [28]. While the limited sample size of paired measurements constrains statistical power, this finding is consistent with pharmacological theory predicting that these endpoints capture fundamentally different aspects of drug action. Our dual-endpoint classification strategy, requiring high predicted potency for both IC50 and EC50 (“High-High”), therefore represents a conservative approach that reduces false-positive prioritization by demanding favorable predictions across both biochemical and cellular dimensions.

The successful application of ECFP4 fingerprints, which captured 844 of the 887 selected features, aligns with their established utility in natural product QSAR studies [43,44], effectively encoding local structural environments that are particularly relevant for natural products’ biological activity.

The performance metrics achieved (MCC = 0.583, ROC-AUC = 0.896) are competitive with state-of-the-art QSAR models for antiviral prediction. For comparison, recent studies targeting flavivirus inhibitors report MCC values ranging from 0.45–0.65 [22,45,46], with our optimized model performing in the upper range. The inclusion of assay type as a distinguishing feature proved crucial, improving MCC by approximately 0.08 compared to models without this distinction. This highlights the importance of considering experimental context in QSAR modeling, particularly when combining data from different assay formats.

The comprehensive structure–activity relationship (SAR) analysis of the 276 High-High compounds revealed critical insights into the molecular determinants of anti-dengue activity and the challenges inherent in natural product drug discovery. The striking disparity in drug-likeness—with only 20 compounds (7.2%) achieving QED ≥ 0.5—reflects a fundamental tension between the evolutionary optimization of phytochemicals for ecological functions versus pharmaceutical requirements for human therapeutics [47]. Natural products have evolved to maximize biological activity through structural features that often violate Lipinski’s Rule of Five [41], including high molecular weight, excessive hydrogen bonding capacity, and elevated polar surface area. Our finding that 256 compounds (92.8%) exhibited suboptimal drug-likeness despite high predicted potency underscores the necessity of multi-criteria filtering in virtual screening campaigns and validates our strategic focus on the 20 high-QED candidates for experimental prioritization.

The remarkable structural novelty observed among high-potency hits—with all 276 compounds (100%) exhibiting Tanimoto similarity < 0.5 to ChEMBL training data (median: 0.214)—provides compelling evidence that Colombian biodiversity harbors unique chemical scaffolds worthy of systematic investigation. Murcko scaffold analysis revealed 145 unique scaffolds, of which 144 (99.3%) were completely absent from the training dataset. This unprecedented novelty rate far exceeds typical virtual screening campaigns against synthetic libraries, where 40–60% novelty is considered exceptional [48,49]. The chemical space segregation visualized through principal component analysis (Figure 3B) confirms that novel candidates occupy distinct structural regions, suggesting that they may engage dengue viral targets through alternative binding modes or access entirely different protein pockets compared to known inhibitors. This structural divergence presents both opportunities and challenges: while novel scaffolds offer potential for circumventing resistance mechanisms and accessing unexplored intellectual property space, they also require more extensive mechanistic characterization and may necessitate greater structural optimization to achieve clinical candidates.

The descriptor–activity correlation analysis (Figure 3D) revealed differential molecular property associations between IC50 and EC50 endpoints, reinforcing their biological distinction. The weak correlations observed for IC50 activity (maximum |ρ| = 0.06) compared to EC50 (maximum |ρ| = 0.15) suggest that biochemical target inhibition is influenced by subtle structural features not captured by simple physicochemical descriptors, potentially involving specific three-dimensional arrangements or pharmacophoric elements. In contrast, the stronger correlations for EC50—particularly the negative TPSA association (ρ = −0.15) and positive QED relationship (ρ = 0.14)—indicate that cellular antiviral efficacy is more predictable from bulk molecular properties, likely reflecting the importance of membrane permeability, intracellular distribution, and metabolic stability. The TPSA-EC50 negative correlation provides actionable guidance for lead optimization: strategic reduction of the polar surface area through modifications such as methylation of hydroxyl groups, replacement of carboxylic acids with bioisosteric alternatives, or removal of sugar moieties from glycosides could enhance cellular penetration while maintaining target affinity. Conversely, the positive QED-EC50 association validates our prioritization strategy, as compounds naturally possessing drug-like features are inherently more likely to demonstrate cellular efficacy without requiring extensive medicinal chemistry optimization.

Detailed examination of the 15 top-priority candidates (Table 2, Figure 4) revealed a structurally diverse collection spanning five chemical classes, with alkaloids predominating (*n* = 7, 46.7%). Incartine (#1), an indole alkaloid from *Hippeastrum puniceum* exhibiting the highest QED score (0.83) and balanced dual-endpoint activity (pIC50: 6.84, pEC50: 6.13), emerged as the most promising lead candidate. Its favorable pharmaceutical profile—moderate molecular weight (312.4 Da), balanced lipophilicity (LogP: 2.1), and low polar surface area (TPSA: 67.8 Ų)—combined with structural novelty (Tanimoto: 0.28) positions it as an ideal starting point for medicinal chemistry optimization. The identification of two compounds from *Hippeastrum puniceum* among the top 15 (Incartine #1 and Hippeastrine #11) suggests that this species may be particularly rich in anti-dengue scaffolds, warranting comprehensive phytochemical profiling and possibly representing an underexplored source of Amaryllidaceae alkaloids with antiviral potential.

Comprehensive ADMET profiling (Figure 6) revealed critical insights into the pharmaceutical viability of priority candidates and exposed a fundamental trade-off between predicted antiviral potency and drug-likeness. While all 15 candidates demonstrated universal compliance with Lipinski’s Rule of Five and absence of PAINS alerts, integrated ADMET scoring identified substantial variation in overall pharmaceutical favorability (scores ranging from 0.629 to 0.825). Notably, Incartine—despite ranking first by predicted pIC50 (6.84)—exhibited only moderate ADMET score (0.698, rank 11/15) due to computational flags for AMES mutagenicity, hepatotoxicity, CYP2D6 inhibition, and hERG II inhibition. This contrasts sharply with Trifolirhizin, which achieved the highest ADMET score (0.825) while maintaining robust predicted activity (pIC50: 6.63). This dissociation between computational potency predictions and pharmaceutical profiles validates our multi-criteria optimization approach and underscores that QSAR-predicted activity alone provides insufficient basis for candidate prioritization. The observation that 80% of candidates flagged positive for AMES mutagenicity, while concerning, must be interpreted cautiously given the documented 35–45% false-positive rate of computational AMES predictions for natural products, necessitating experimental validation through bacterial reverse mutation assays. The structural class-dependent ADMET patterns observed—with terpenoid glycosides (mean score: 0.785) consistently outperforming Amaryllidaceae alkaloids (mean score: 0.709) in safety and metabolic stability predictions—provides actionable guidance for future bioprospecting efforts and suggests that the systematic investigation of terpenoid-rich Colombian plant families may yield inherently more pharmaceutically tractable candidates.

The presence of Lycorine (13, Tanimoto: 1.00) and Pseudolycorine (15, Tanimoto: 1.00) among our top predictions provides crucial internal validation of model performance. These compounds, present in the ChEMBL training dataset with experimentally confirmed anti-dengue activity, were successfully recovered by our screening protocol despite not being explicitly identified as validation targets. Lycorine, in particular, has demonstrated broad-spectrum antiviral activity in recent studies, including against SARS-CoV-2 and influenza A virus through mechanisms involving protein synthesis inhibition and immunomodulation [50,51]. The model’s ability to correctly prioritize these validated compounds increases confidence in predictions for structurally novel candidates and suggests that our top 12 novel compounds merit immediate experimental evaluation.

Intriguingly, several terpenoid glycosides appeared among the top candidates (Byzantionoside B #5, (6R,9R)-3-Oxo-alpha-ionol glucoside #10, 9-Hydroxy-7-megastigmen-3-one glucoside #12), despite their typically challenging pharmaceutical properties. Glycosylation, while often improving water solubility and reducing toxicity, generally compromises oral bioavailability due to poor membrane permeability and susceptibility to intestinal glycosidases [52]. However, for dengue treatment—where severe cases require hospitalization and intravenous administration is standard—this limitation may be less critical than for orally administered therapeutics. Additionally, prodrug strategies employing enzymatically cleavable linkers could enable the targeted delivery of aglycone active compounds while exploiting the favorable solubility of glycosides. The moderate QED scores achieved by these glycosides (0.51–0.54) suggest that they occupy a pharmaceutical property space that, while suboptimal for oral drugs, may be acceptable for parenteral formulations or with appropriate delivery modifications.

Comparative analysis with ChEMBL training set high-potency references (Figure 4, right panels) revealed both validating similarities and important distinctions. The training set was dominated by Amaryllidaceae alkaloids, with Narciclasine analogs (ChEMBL464432, pActivity: 10.82) and Pancratistatin (pActivity: 10.20) exhibiting exceptional nanomolar potencies (IC50 = 0.01–0.06 nM). However, many of these highly potent training compounds exhibit low drug-likeness (e.g., ChEMBL5188858 QED: 0.22, ChEMBL3392006 QED: 0.13), illustrating the disconnect between biochemical potency and pharmaceutical viability that our filtering strategy explicitly addresses. Our identification of structurally related but drug-like Amaryllidaceae alkaloids (Incartine QED: 0.83, 3-Acetylnerbowdine QED: 0.80) represents a potential breakthrough in balancing potency with developability.

The structural diversity among our novel candidates—ranging from simple indole alkaloids (Incartine, Indican) to complex sesquiterpene lactones (Bilobalide, Epicolactone) to cardiac glycoside aglycones (Digoxigenin)—contrasts sharply with the training set’s heavy enrichment in Amaryllidaceae alkaloids. This chemical diversity suggests our top candidates may target multiple points in the dengue viral replication cycle or engage different protein pockets, offering opportunities for rational combination therapy. Recent structural biology studies have identified multiple druggable sites across dengue viral proteins, including the NS3 protease active site [53], NS5 RNA-dependent RNA polymerase catalytic pocket [54], NS2B-NS3 protease allosteric sites [55], and envelope protein domain II [56]. The structural heterogeneity of our candidates makes it plausible that different chemical classes engage different targets, potentially enabling synergistic multi-target inhibition strategies that could address antiviral resistance.

The finding that only 14.3% of Colombian plant species have documented antiviral activity, and nearly half of these lack phytochemical characterization, reveals both a challenge and an opportunity. This knowledge gap is particularly concerning given Colombia’s status as the world’s second-most biodiverse country [57] and the increasing pressure on natural habitats from deforestation and climate change [58]. The urgency to document and preserve this chemical diversity cannot be overstated, as each lost species potentially represents unique bioactive compounds that could address current and future health challenges.

The predominance of certain plant families in our antiviral activity compilation—particularly Asteraceae, Fabaceae, and Lamiaceae—reflects both their chemical richness and the historical focus of ethnopharmacological research. Asteraceae’s prevalence is unsurprising given its production of diverse sesquiterpene lactones, compounds with well-documented antiviral properties [59,60]. However, this distribution may also reflect sampling bias, as these families are among the most studied globally, potentially overlooking equally valuable but less investigated families. However, this distribution may also reflect sampling bias, as these families are among the most studied globally, potentially overlooking equally valuable but less investigated families. Our identification of Digoxigenin (#14), a cardiac glycoside aglycone from Digitalis purpurea, as a high-priority candidate despite steroids being underrepresented in anti-dengue literature, exemplifies how unbiased computational screening can reveal unexpected chemotype–activity relationships that might be missed by hypothesis-driven approaches.

The identification of Amaryllidaceae alkaloids—specifically Incartine, Lycorine, and Kalbreclasine from *Hippeastrum puniceum*—as top candidates provides compelling validation of our approach. Lycorine has demonstrated broad-spectrum antiviral activity in recent studies, including against SARS-CoV-2 [50] and influenza viruses [51], through mechanisms involving protein synthesis inhibition and immunomodulation. The high predicted activity of these compounds against dengue virus (pActivity > 7.0) suggests potential for repurposing these natural products, which could significantly accelerate drug development timelines. The structural analysis revealing that 100% of the 276 High-High compounds represent novel chemotypes (Tanimoto < 0.5) not present in the ChEMBL training set is particularly significant. This finding suggests that Colombian flora contains a unique structural diversity that could provide new mechanisms of action against dengue virus. Among the 15 prioritized candidates, alkaloids predominate (7/15, 46.7%), followed by terpenoids (6/15, 40.0%), flavonoids (1/15, 6.7%), and steroids (1/15, 6.7%). This chemical diversity contrasts with current anti-dengue drug development, which has primarily focused on nucleoside analogs and protease inhibitors.

The favorable drug-likeness profiles of our 15 prioritized candidates—all satisfying Lipinski’s Rule of Five with zero violations and QED scores ranging from 0.51 to 0.83—suggest good potential for pharmaceutical development. However, the presence of glycosylated compounds (5/15, 33.3%) presents both opportunities and challenges. While glycosylation can improve water solubility and reduce toxicity, it often compromises oral bioavailability due to poor membrane permeability and susceptibility to intestinal glycosidases. The alignment between our computational predictions and traditional uses of these plants provides important validation. 

This study has several limitations that should be considered when interpreting the results:

Model-related limitations: The QSAR model’s applicability domain may not fully encompass the structural complexity of natural products, particularly those with multiple chiral centers or unusual functional groups rare in synthetic compounds. While our model achieved competitive performance (MCC = 0.583, ROC-AUC = 0.896), predictions for highly novel compounds (Tanimoto < 0.3) should be interpreted with appropriate caution.

Activity threshold considerations: The potency thresholds employed (pActivity > 6 for high potency) follow established drug discovery conventions for submicromolar activity classification [61,62]. Sensitivity analysis demonstrated that compounds classified as high potency in the training dataset exhibited pActivity values substantially above the threshold (mean: 7.04 ± 0.97; margin: +1.04 log units). The prioritized candidates exhibited predicted pActivity values above the cutoff (mean pIC_50_: 6.42 ± 0.30; mean pEC_50_: 6.45 ± 0.34), with margins (+0.42 to +0.45 log units) consistent with expected QSAR model uncertainty (±0.3–0.5 log units) and experimental variability in heterogeneous IC_50_ data (σ = 0.68) [63]. The dual-endpoint “High-High” classification requirement provides additional conservatism against threshold-dependent artifacts.

Training dataset considerations: The training dataset exhibited class imbalance characteristic of bioactivity databases, with high-potency compounds representing 11.1% of the data. While this imbalance was addressed through sample weighting mechanisms and validated using appropriate metrics (MCC, ROC-AUC), the limited sample size of high-potency examples (*n* = 227) may constrain the model’s ability to capture the full structural diversity of potent inhibitors. The dataset size of 2034 compounds represents the maximum available high-confidence experimental data for dengue virus targets in public databases [64,65].

Experimental validation: This study represents an in silico screening campaign without experimental validation of predicted activities. While computational predictions provide valuable prioritization, in vitro and in vivo studies are necessary to confirm antiviral efficacy, determine mechanisms of action, and assess safety profiles.

Single-compound approach: Our methodology evaluated individual compounds in isolation, whereas traditional medicinal preparations often rely on synergistic interactions between multiple constituents. The potential for synergistic or antagonistic effects among phytochemicals was not assessed.

Mechanistic insights: The lack of target-specific predictions represents a limitation; while our model predicts general anti-dengue activity, it does not identify which viral proteins are targeted, which would greatly facilitate lead optimization. As a ligand-based QSAR approach, this study predicts general anti-dengue activity without identifying specific molecular targets. The IC50 and EC50 measurements used for model training derive from whole-virus assays that may reflect the inhibition of multiple viral proteins or host–pathogen interactions. Consequently, structure-based computational methods such as molecular docking and molecular dynamics simulations are not appropriate at this stage, as they require prior knowledge of the specific protein target(s). Target deconvolution studies using experimental approaches (thermal shift assays, enzymatic inhibition assays, or surface plasmon resonance) represent essential next steps following vitro validation of antiviral activity and would enable subsequent structure-based optimization of confirmed hits.

Data availability and sampling bias: The 48.6% of antiviral plant species lacking phytochemical characterization represents a significant knowledge gap that may cause us to overlook potentially important bioactive compounds from understudied species. Additionally, the literature-based screening for antiviral activity inherently favors well-studied cosmopolitan families (e.g., Asteraceae, Fabaceae, Lamiaceae) over endemic or understudied taxa. This reporting bias likely results in conservative underestimation of Colombian flora’s true antiviral potential rather than the inflation of results, as species lacking published antiviral studies were excluded regardless of their potential bioactivity.

Despite these limitations, this computational framework provides a valuable prioritization tool that can significantly reduce the time and cost of experimental screening. The public availability of our model and datasets enables other researchers to validate predictions, extend the analysis to additional plant species, or adapt the methodology for other therapeutic targets.

This study’s implications extend beyond immediate drug discovery applications. By demonstrating the pharmaceutical potential of Colombian biodiversity, we provide economic arguments for conservation that complement ethical and ecological rationales. The identification of high-value compounds from native species could support sustainable development models where local communities benefit from biodiversity conservation through benefit-sharing agreements aligned with the Nagoya Protocol. The development of an open-access platform for continuing this screening effort democratizes access to these findings and enables collaborative research, aligning with global efforts to make drug discovery more inclusive. This pioneering work successfully demonstrates the feasibility and value of applying modern computational approaches to explore Colombian biodiversity for anti-dengue drug discovery. Moving forward, immediate priorities include experimental validation of top candidates through viral inhibition assays, mechanism of action studies for confirmed hits, and chemical characterization of the understudied antiviral plant species. This integrated approach positions Colombia to leverage its extraordinary biodiversity for global health benefit while promoting the sustainable development and conservation of its natural heritage.

## 4. Materials and Methods

### 4.1. Data Sources and Availability

All datasets, trained models, and analysis code generated during this study are publicly available to ensure full reproducibility. Bioactivity Data Raw anti-dengue bioactivity data were retrieved from the ChEMBL database, version 33 [31,66] (https://www.ebi.ac.uk/chembl/, accessed 1 March 2024). The curated training dataset is provided as Data S1 in the Supplementary Materials. Plant species information was obtained from the official checklist ‘Plantas alimenticias y medicinales de Colombia’ (I2D-BIO_2014_IN045), a Darwin Core-standardized dataset curated by the Alexander von Humboldt Biological Resources Research Institute covering the entirety of Colombian territory [11]. The complete Colombian phytochemical library (3267 compounds from 358 medicinal plant species) is available as Data S2 in the Supplementary Materials. Detailed ethnobotanical information for all plant species is provided in Table S1. Computational Models and Code. The optimized XGBoost model, preprocessing objects, and model parameters are available as Data S3 in the Supplementary Materials. All Python scripts for data curation, molecular descriptor calculation, model training, Bayesian optimization, and virtual screening are deposited at https://github.com/Sergio111999/QSAR_DENV#qsar-denv-anti-dengue-drug-discovery-from-colombian-medicinal-flora (accessed on 1 November 2025) under an MIT license (DOI to be provided upon acceptance).

### 4.2. Anti-Dengue Compound Dataset Curation

Bioactivity data were retrieved from ChEMBL using the following SQL query structure:

sqlSELECT * FROM activities

WHERE target_organism LIKE ‘%dengue%’

AND standard_type IN (‘IC50’, ‘EC50’)

AND standard_relation = ‘=’

Data curation followed the workflow described by Mendez et al. (2019) [31]. Briefly, activity values were standardized to molar units and converted to pActivity (−log10[M]). Compounds were categorized into three potency classes following the established drug discovery conventions: high potency (pActivity > 6, corresponding to submicromolar activity IC50/EC50 < 1 µM), medium potency (5 < pActivity ≤ 6, corresponding to low micromolar activity 1–10 µM), and low potency (pActivity ≤ 5, corresponding to IC50/EC50 > 10 µM). The pActivity > 6 threshold for high potency represents the widely accepted benchmark for identifying compounds with sufficient potency to warrant further development in drug discovery campaigns [61,62]. Duplicate structures were identified using InChIKey and resolved by retaining the highest pChEMBL value. The final dataset contained 2034 activity records corresponding to 1981 unique molecules, with 53 molecules having both IC50 and EC50 measurements.

IC_50_ and EC_50_ endpoints were modeled independently based on three considerations: (1) pharmacological distinction—IC_50_ measures biochemical target inhibition while EC_50_ integrates cellular factors including membrane permeability, metabolic stability, and intracellular distribution; (2) statistical independence—analysis of 53 compounds with both measurements revealed no significant correlation (r = −0.0415, *p* = 0.77, 95% CI: [−0.31, 0.23]); and (3) data utilization—independent modeling preserves 1339 IC_50_ and 695 EC_50_ measurements that would be lost if analysis was restricted to the 53-compound overlap.

### 4.3. Colombian Medicinal Flora Database Construction

The initial species pool (*n* = 2567) was obtained from the official checklist ‘Plantas alimenticias y medicinales de Colombia’ (I2D-BIO_2014_IN045), curated by the Alexander von Humboldt Biological Resources Research Institute through systematic review of Colombian herbaria with taxonomic validation against the Missouri Botanical Garden database (Tropicos) [11]. This Darwin Core-standardized dataset provides nationwide geographic coverage and was developed under Colombia’s National Strategy for Plant Conservation.

After removing duplicate entries and infraspecific taxa, 2501 unique species remained for analysis. Species with documented antiviral activity were identified through a systematic literature review following the PRISMA guidelines [67]. PubMed and Scopus were searched (January–April 2024) using: “[species name]” AND “antiviral”. Of the 2501 species evaluated, 358 (14.3%) exhibited documented antiviral activity. However, within this subset, only 184 species (51.4%) had any level of phytochemical characterization or reported metabolites in the literature to which the observed antiviral activity could be attributed. The structures of these metabolites were retrieved from the primary literature and standardized using RDKit (v2024.03.5), following the protocol of Bento et al. (2020) [68].

### 4.4. Molecular Descriptors and Machine Learning

Molecular descriptors (*n* = 43) and fingerprints (ECFP4, 2048 bits) were calculated using RDKit. The complete feature matrix (2091 features) was reduced to 887 informative features using variance filtering (threshold > 0.01). Correlation analysis of the 40 physicochemical descriptors identified clusters of related features (e.g., molecular size indices: MW, Chi0, Chi1, MolMR), which is expected given their chemical relationships. However, these correlated descriptors contributed only 6.3% of total model importance, with 91.9% derived from ECFP4 fingerprint bits that lack systematic correlations. Tree-based ensemble methods are inherently robust to multicollinearity for prediction tasks, as correlated features compete for split selection without affecting predictive performance.

Machine learning models were implemented using scikit-learn (v1.3.2) and XGBoost (v3.0.2). To address class imbalance in the training dataset (High potency: 11.1%; Medium: 23.4%; Low: 65.5%), sample weights were calculated during XGBoost training using the formula weight[class] = N_total/(N_classes × N_class). This approach weights minority class instances proportionally to their underrepresentation, ensuring that classification errors on high-potency compounds contribute appropriately to model updates. Additional protection against overfitting was achieved through XGBoost’s comprehensive regularization: L1 regularization (reg_alpha = 0.016), L2 regularization (reg_lambda = 4.73), tree pruning (gamma = 1.53), and column subsampling (colsample_bytree = 0.91).

Model performance was evaluated using the Matthews Correlation Coefficient (MCC) and ROC-AUC, metrics specifically suited for imbalanced classification. Model evaluation employed 5-fold stratified cross-validation. The optimal model (XGBoost with Descriptors+ECFP4 representation) achieved an MCC = 0.572 ± 0.048.

#### Model Validation

Model validation employed four complementary approaches to ensure robustness and prevent overfitting:

Extended Bayesian Optimization: Hyperparameter optimization was extended to 100 trials to confirm convergence. Analysis revealed that 99.6% of the final performance was achieved by trial 50 (MCC = 0.5832 vs. final 0.5854), with the marginal improvement of 0.38% demonstrating adequate convergence within the original optimization budget.

Y-Randomization Testing: To validate that the model captures genuine structure-activity relationships rather than random correlations, Y-randomization testing was performed with 100 permutations of the target labels. The real model MCC (0.585) was dramatically higher than any permuted model (max = 0.059, *p* < 0.01), confirming that the model captures genuine structure–activity relationships.

Hyperparameter Importance Analysis: Regularization parameters dominated the optimization landscape: reg_alpha (38.6%), gamma (33.3%), and reg_lambda (0.8%), collectively accounting for 72.7% of performance variance, indicating the optimization process prioritized overfitting prevention.

Independent Hold-out Validation: A stratified 80/20 train–test split with completely unseen test data (*n* = 407) was performed. Hold-out MCC (0.576) differed by only 1.2% from CV MCC (0.583), and hold-out ROC-AUC (0.888) differed by only 0.9% from CV ROC-AUC (0.896), confirming that cross-validation estimates accurately predict performance on completely unseen data.

### 4.5. Virtual Screening Protocol

The trained XGBoost model was applied to predict anti-dengue activity for 3267 phytochemicals from Colombian medicinal flora. For each compound, predictions were generated independently for both IC50 and EC50 endpoints, yielding pActivity values calculated as −log10 (predicted activity in molar units). The dual-endpoint classification framework categorized compounds into nine classes based on combined IC50 and EC50 potency thresholds: Low (pActivity ≤ 5.0), Medium (5.0 < pActivity ≤ 6.0), and High (pActivity > 6.0). Compounds achieving high predicted potency for both endpoints (pIC50 > 6.0 AND pEC50 > 6.0) were designated as “High-High” and prioritized for subsequent analysis.

Drug-likeness assessment was performed using Lipinski’s Rule of Five criteria and Quantitative Estimate of Drug-likeness (QED) scores calculated following the methodology of Bickerton et al. [47] as implemented in RDKit. QED integrates eight molecular properties (MW, LogP, HBA, HBD, PSA, rotatable bonds, aromatic rings, structural alerts) into a unified drug-likeness metric ranging from 0 (non-drug-like) to 1 (ideal drug-like properties). A QED threshold of 0.5 was applied to distinguish compounds with favorable pharmaceutical properties from those requiring extensive optimization.

Compounds were prioritized for experimental validation based on a multi-criteria strategy: (i) dual high potency (pActivity > 6.0 for both IC50 and EC50), (ii) favorable drug-likeness (QED ≥ 0.5), (iii) structural novelty assessment via Tanimoto similarity to training compounds, and (iv) compliance with applicability domain criteria. This hierarchical filtering identified 20 compounds meeting all criteria, from which the top 15 were selected based on pIC50 ranking for detailed characterization.

#### Comprehensive Structure–Activity Relationship (SAR) Analysis

Following QSAR-based virtual screening, a systematic SAR analysis pipeline was implemented to characterize activity distributions, assess chemical diversity, evaluate structural novelty, and identify molecular properties correlated with antiviral potency. The analysis comprised four integrated components executed using Python 3.12.3 with RDKit 2024.03.5, scikit-learn 1.3.2, and custom analysis scripts.

Dual-Endpoint Classification and Activity Distribution: All 3267 screened compounds were classified into a 3 × 3 activity matrix based on combined IC50 and EC50 predicted potencies using the thresholds defined above (Low ≤ 5.0, Medium 5.0–6.0, High > 6.0). This classification generated nine mutually exclusive categories (e.g., “Low-Low “, “High-Medium”, “High-High”), enabling systematic characterization of dual-endpoint activity patterns across the chemical library. Frequency distributions were calculated for each category, and the “High-High” class was designated as the priority population for downstream analyses.

Structural Novelty Assessment: The structural novelty of high-potency compounds relative to the ChEMBL training dataset was quantified using Tanimoto similarity coefficients calculated from Extended Connectivity Fingerprints (ECFP4, radius = 2, 2048 bits) as implemented in RDKit. For each screened compound, Tanimoto similarity was computed against all 1981 training set compounds, and the maximum similarity value was retained as the novelty metric. Compounds were classified into novelty categories as follows: Novel/Very Novel (Tanimoto < 0.5), indicating low structural similarity to known anti-dengue agents; Moderate similarity (0.5 ≤ Tanimoto < 0.7); and Training-like (Tanimoto ≥ 0.7), indicating high structural resemblance to training compounds. This classification enables the identification of both validated chemotypes (high Tanimoto) and potentially innovative scaffolds (low Tanimoto).

Chemical Space Visualization and Diversity Analysis: Principal component analysis (PCA) was performed on the ECFP4 fingerprint matrix to project the high-dimensional chemical space into two dimensions for visualization. PCA was implemented using scikit-learn’s decomposition module with standardized features, and the first two principal components (PC1, PC2) were retained for plotting. The projection enables the visual assessment of chemical space coverage, identification of structural clusters, and evaluation of segregation between novelty categories. The cumulative variance explained by PC1 and PC2 quantifies the effectiveness of dimensionality reduction.

Drug-likeness Assessment: All compounds were evaluated for drug-likeness using the QED metric calculated via RDKit’s QED module (Chem.QED.qed function). QED scores were dichotomized using a threshold of 0.5: compounds with QED ≥ 0.5 were classified as “High QED” (favorable pharmaceutical properties), while QED < 0.5 indicated “Low QED” (suboptimal properties requiring optimization). For High-High compounds, the proportion achieving high QED was calculated to assess the pharmaceutical development challenge inherent in the high-potency population.

Descriptor-Activity Correlation Analysis: Spearman rank correlation coefficients (ρ) were calculated between 11 molecular descriptors and predicted pActivity values for both IC50 and EC50 endpoints across all 3267 compounds. The descriptor set included: Molecular Weight (MW), octanol-water partition coefficient (LogP), hydrogen bond acceptors (HBA), hydrogen bond donors (HBD), rotatable bonds (RotBonds), topological polar surface area (TPSA), total ring count (Rings), aromatic rings (AromaticRings), aliphatic rings (NumAliphaticRings), fraction of sp3 carbons (FractionCSP3), and QED. Correlations were computed using scipy.stats.spearmanr, and statistical significance was assessed using two-tailed tests with Bonferroni correction for multiple comparisons (α = 0.05/22 = 0.0023, accounting for 11 descriptors × 2 endpoints). Only correlations with |ρ| > 0.05 and corrected *p* < 0.0023 were considered statistically significant. Results were visualized as heatmaps using seaborn 0.13.2 with diverging color scales centered at zero correlation.

Top Candidate Selection and Characterization: From the 20 compounds meeting all filtering criteria (High-High + QED ≥ 0.5), the top 15 were selected based on descending pIC50 values and subjected to comprehensive characterization. For each compound, the following properties were tabulated: predicted pIC50 and pEC50, QED score, molecular descriptors (MW, LogP, HBA, HBD, TPSA), novelty category, maximum Tanimoto similarity to training compounds, assigned chemical class based on structural features, and botanical source from phytochemical databases. Chemical class assignments integrated automated substructure searches (using RDKit’s HasSubstructMatch for scaffold identification) with manual curation based on established phytochemical taxonomy. Plant source assignments were cross-referenced with the primary literature and validated against the Colombian medicinal flora database compiled in this study.

All SAR analysis workflows, including data preprocessing, statistical calculations, and visualization generation, were implemented in reproducible Python scripts with full parameterization, enabling adaptation to alternative screening datasets. Complete code with detailed documentation is available in the public repository accompanying this manuscript.

### 4.6. Applicability Domain Assessment

To ensure the reliability of predictions, the applicability domain was evaluated using:

- Chemical similarity: Tanimoto coefficient > 0.3 to nearest training set compounds;
- Leverage analysis: Compounds with leverage hi < 3p/*n* (p = features, *n* = training samples);
- Descriptor range: All molecular descriptors within training set min-max bounds.

Only compounds meeting at least two criteria were considered reliable predictions.

Additionally, leverage analysis was performed by calculating the hat matrix diagonal elements (hi) for each prediction using SVD decomposition for numerical stability, with the warning threshold set at h* = 3p/*n* where p is the number of features (887) and n is the training set size (2034), yielding a critical leverage value of 1.308. Compounds exceeding this threshold were flagged as requiring extrapolation and treated with appropriate caution in interpretation.

Comprehensive applicability domain assessment of the 15 prioritized candidates employed dual criteria following the OECD guidelines. Leverage-based analysis identified 2/15 compounds (13.3%) within the leverage-based AD: Lycorine and Pseudolycorine, both present in the ChEMBL training dataset with experimentally confirmed anti-dengue activity. Tanimoto similarity analysis (threshold > 0.3) identified 5/15 compounds (33.3%) within acceptable structural coverage: Lycorine (Tc = 1.000), Pseudolycorine (Tc = 1.000), Hippeastrine (Tc = 0.515), Indican (Tc = 0.345), and Incartine (Tc = 0.328). The observation that most prioritized compounds fell outside the leverage-based AD was scientifically expected for natural products from underexplored chemical space and does not invalidate the predictions, as these compounds represent the novel chemotypes that constitute the primary objective of this bioprospecting study. Based on AD assessment, experimental validation is recommended in three tiers: Tier 1 (highest confidence): Lycorine, Pseudolycorine—within both AD criteria; Tier 2 (high confidence): Hippeastrine, Indican, Incartine—within Tanimoto AD; Tier 3 (exploratory): remaining compounds—novel chemotypes requiring experimental confirmation.

### 4.7. ADMET Profiling of Prioritized Candidates

The 15 prioritized compounds from virtual screening were subjected to comprehensive in silico ADMET (Absorption, Distribution, Metabolism, Excretion, and Toxicity) profiling using a multi-platform approach to assess pharmaceutical viability prior to experimental validation.

Physicochemical properties and pharmacokinetic parameters were computed using SwissADME [69], which calculated molecular descriptors including molecular weight (MW), lipophilicity (consensus Log P from five independent methods: iLOGP, XLOGP3, WLOGP, MLOGP, Silicos-IT), topological polar surface area (TPSA), hydrogen bond acceptors/donors (HBA/HBD), number of rotatable bonds, molar refractivity (MR), and fraction of sp3 carbons (Csp3). Absorption properties evaluated included gastrointestinal (GI) absorption level (High/Low classification), and blood–brain barrier (BBB) permeability (Yes/No). Distribution parameters included P-glycoprotein (P-gp) substrate status and skin permeability coefficient (log Kp). Metabolic liability was assessed through the prediction of inhibition for five major cytochrome P450 isoforms (CYP1A2, CYP2C9, CYP2C19, CYP2D6, CYP3A4), which collectively mediate >80% of Phase I drug metabolism. Solubility was predicted using three independent algorithms (ESOL, Ali, Silicos-IT), with the ESOL classification (Very soluble/Soluble/Moderately soluble/Poorly soluble/Insoluble) used as the primary descriptor.

Drug-likeness was evaluated according to established medicinal chemistry filters: Lipinski’s Rule of Five (MW ≤ 500 Da, Log P ≤ 5, HBA ≤ 10, HBD ≤ 5), Ghose filter (160 ≤ MW ≤ 480, −0.4 ≤ Log P ≤ 5.6, 40 ≤ MR ≤ 130, 20 ≤ atoms ≤ 70), Veber rule (rotatable bonds ≤ 10, TPSA ≤ 140 Ų), Egan rule (TPSA ≤ 131.6 Ų, Log P ≤ 5.88), and Muegge rule. Structural alerts were evaluated for pan-assay interference compounds (PAINS) using established substructure filters and for potentially reactive or toxic functional groups (Brenk alerts). Bioavailability scores were computed as the probability of achieving ≥10% oral bioavailability in rats. Synthetic accessibility was assessed on a 1–10 scale (lower scores indicating easier synthesis) using fragment-based analysis of structural complexity.

Toxicological endpoints were predicted using pkCSM [70], which provides empirical models trained on experimental data for: (1) Ames mutagenicity (bacterial reverse mutation assay), (2) hERG I and II cardiac ion channel inhibition as cardiotoxicity surrogates, (3) hepatotoxicity, (4) skin sensitization potential, (5) oral rat acute toxicity (LD50 in mol/kg), (6) oral rat chronic toxicity (LOAEL in log mg/kg_bw/day), and (7) maximum tolerated dose in humans (log mg/kg/day). Environmental toxicity was additionally assessed through Tetrahymena pyriformis and fathead minnow (*Pimephales promelas*) toxicity predictions.

An integrated ADMET favorability scoring system was developed by converting each property to a normalized 0–1 scale where 1.0 represents the optimal pharmaceutical properties and 0.0 represents unfavorable characteristics. The conversion scheme was property-specific: (1) for binary categorical properties (e.g., GI absorption: High = 1.0, Low = 0.0; BBB permeant: No = 1.0 for peripheral drugs, Yes = 0.0), (2) for ordinal solubility classes (Very soluble = 1.0, Soluble = 0.75, Moderately soluble = 0.5, Poorly soluble = 0.25), (3) for continuous toxicity values, normalization by min-max scaling with higher LD50 values corresponding to higher favorability scores, (4) for synthetic accessibility, inversion of the 1–10 scale such that easier synthesis received higher scores, and (5) for violation counts, inverse scoring (1.0—violations/max_violations). The global ADMET score for each compound was calculated as the arithmetic mean across all 17 normalized properties. Hierarchical clustering of compounds based on their ADMET profiles was performed using Ward’s method with Euclidean distance on the complete favorability score matrix. Statistical analyses were conducted in Python 3.12.3 using pandas, NumPy, SciPy, and scikit-learn libraries. Heatmap visualizations were generated using Matplotlib and Seaborn with a diverging color scheme (green-yellow-red) to facilitate interpretation of favorable versus unfavorable properties.

### 4.8. Statistical Analysis

Statistical analyses used Python 3.12.3. Distribution comparisons employed the Mann–Whitney U test. Multiple testing correction used the Bonferroni method (α = 0.05). Chemical space visualization used UMAP (umap-learn v0.5.7; n_neighbors = 15, min_dist = 0.1) and hierarchical clustering (Ward linkage on Tanimoto distances).

### 4.9. Computational Resources

All computations were performed on a workstation equipped with an Intel Core i5 CPU, 16 GB RAM, SSD storage, and dual GPUs (NVIDIA GeForce RTX 3050 Laptop GPU with 4 GB dedicated VRAM and Intel Iris Xe Graphics), running Windows 11. XGBoost and LightGBM models utilized GPU acceleration via CUDA support. Model training required approximately 6 h, while the virtual screening of 3267 compounds was completed in three minutes.

### 4.10. Code Availability

Complete code for reproducing all analyses is available at https://github.com/Sergio111999/QSAR_DENV#qsar-denv-anti-dengue-drug-discovery-from-colombian-medicinal-flora under MIT license. Key dependencies: Python (3.12.3) [71], RDKit (2024.03.5) [72], NumPy (1.26.4) [73], pandas (2.3.0) [74], SciPy (1.13.1) [75], scikit-learn (1.3.2) [76], XGBoost (3.0.2) [19], LightGBM (4.6.0) [77], Optuna (4.4.0) [29], imbalanced-learn (0.12.3) [78], matplotlib (3.9.2) [79], seaborn (0.13.2) [80], joblib (1.4.2), umap-learn (0.5.7) [32], streamlit (1.37.1) [81].

### 4.11. Use of AI Tools

Claude Opus 4.1 was used to assist with the code debugging and optimization of data visualization scripts. All AI-generated code was manually reviewed and validated. No AI tools were used for data generation, analysis, or interpretation of results.

## 5. Conclusions

This study successfully demonstrated the application of optimized machine learning-based QSAR modeling integrated with comprehensive structure–activity relationship analysis to systematically screen Colombian medicinal flora for potential anti-dengue compounds, revealing the untapped pharmaceutical potential of one of the world’s most biodiverse regions. The development of a robust predictive model through Bayesian hyperparameter optimization (MCC = 0.583, ROC-AUC = 0.896) enabled the virtual screening of 3267 phytochemicals, identifying 276 compounds (8.4%) with dual high potency (High-High classification) and subsequently prioritizing 15 exceptional candidates combining high predicted activity, favorable drug-likeness (QED ≥ 0.5), and diverse structural scaffolds for experimental validation.

The comprehensive SAR analysis revealed critical insights into the molecular landscape of anti-dengue activity within Colombian biodiversity. All 276 High-High compounds (100%) exhibited Tanimoto similarity < 0.5 to ChEMBL training compounds (median: 0.214), representing 145 unique Murcko scaffolds of which 144 (99.3%) were absent from the training data. This demonstrates that Colombian flora harbors unique chemical scaffolds potentially operating through mechanisms distinct from known anti-dengue agents. This exceptionally high novelty rate—substantially exceeding typical synthetic library screening campaigns—positions Colombia as a potential leader in natural product-based antiviral discovery and highlights the importance of biodiversity conservation for pharmaceutical innovation. Principal component analysis confirmed clear chemical space segregation between novel and training-like compounds, validating the structural distinctiveness of prioritized candidates and suggesting that they may access alternative binding modes or target different viral proteins compared to established inhibitors.

The striking disparity in drug-likeness among high-potency compounds—with only 20 candidates (7.2% of High-Highhits) achieving QED ≥ 0.5—illuminates a fundamental challenge in natural product drug discovery: the inherent tension between evolutionary optimization for biological activity versus pharmaceutical requirements for human therapeutics. This finding underscores the critical importance of multi-criteria optimization strategies and validates our hierarchical filtering approach. Descriptor–activity correlation analysis provided actionable mechanistic insights, revealing that QED positively correlates with EC50 activity (ρ = 0.14), while TPSA negatively correlates (ρ = −0.15), suggesting that moderate polarity optimization and inherent drug-like features favor cellular antiviral efficacy. The differential correlation patterns between IC50 (maximum |ρ| = 0.06) and EC50 (maximum |ρ| = 0.15) endpoints validate the biological distinction between biochemical target inhibition and cellular efficacy, reinforcing the value of our dual-endpoint classification strategy.

The identification of Incartine from *Hippeastrum puniceum* as the top candidate, exhibiting the highest QED score (0.83) among all prioritized compounds with balanced dual-endpoint activity (pIC50: 6.84, pEC50: 6.13) and structural novelty (Tanimoto: 0.28), provides a compelling lead for immediate experimental validation. The structural diversity among the top 15 candidates—spanning five chemical classes including alkaloids (46.7%), terpenoid glycosides (26.7%), terpenoids (13.3%), flavonoids (6.7%), and steroids (6.7%)—suggests multiple potential mechanisms of antiviral action and offers opportunities for rational combination therapy strategies. Notably, the presence of Lycorine and Pseudolycorine (both with Tanimoto = 1.00) among the top predictions provides crucial internal validation, as these compounds are present in the ChEMBL training dataset with experimentally confirmed anti-dengue activity, increasing confidence in predictions for structurally novel candidates.

The enrichment of Amaryllidaceae alkaloids among the top candidates—with *Hippeastrum puniceum* contributing two compounds (Incartine #1, Hippeastrine #11) and the family collectively providing four of fifteen prioritized candidates—suggests that this botanical family harbors particularly rich anti-dengue chemical space warranting systematic phytochemical investigation. This finding aligns with emerging evidence of broad-spectrum antiviral properties in *Amaryllidaceae* alkaloids, exemplified by Lycorine’s demonstrated activity against SARS-CoV-2, influenza, and dengue viruses. The convergence between our computational predictions and traditional medicinal uses, exemplified by the identification of compounds from plants like Psidium guajava and *Indigofera suffruticosa* with documented ethnopharmacological applications in dengue-endemic regions, underscores the value of integrating indigenous knowledge with modern drug discovery approaches.

Our analysis revealed a critical knowledge gap in Colombian biodiversity research, with 48.6% of the 358 plant species showing documented antiviral activity lacking phytochemical characterization. This represents both an urgent conservation priority and a substantial opportunity for future drug discovery efforts. As Colombia faces dual challenges of increasing dengue burden and accelerating biodiversity loss through deforestation and climate change, the urgency to document and preserve this chemical diversity cannot be overstated. Each lost species potentially represents unique bioactive compounds that could address current and future health challenges, making biodiversity conservation not only an ecological imperative but also a strategic pharmaceutical resource.

The optimization process yielded important methodological insights beyond the immediate anti-dengue application. The 1.9% improvement in MCC through Bayesian optimization, achieved primarily through regularization parameter tuning (reg_alpha: 38.6%, gamma: 33.3% importance), highlights the critical role of controlling model complexity in a high-dimensional chemical space. The decision to select the optimized XGBoost model over the marginally better ensemble (MCC difference = 0.001) exemplifies the practical trade-offs between performance and complexity in applied QSAR modeling, demonstrating that sophisticated ensemble methods may not always justify their computational overhead. These methodological lessons are transferable to other drug discovery campaigns and contribute to the growing body of best practices in computational chemoinformatics.

The tiered prioritization strategy developed in this study—combining dual-endpoint classification, drug-likeness filtering, novelty assessment, and applicability domain validation—provides a replicable framework for natural product virtual screening campaigns. This methodology addresses key limitations of traditional QSAR approaches by explicitly considering multiple pharmacological dimensions, pharmaceutical developability, and prediction reliability. The framework’s modular design enables adaptation to alternative therapeutic targets, different chemical libraries, and varied experimental validation capacities, making it broadly applicable beyond the immediate anti-dengue context.

Immediate research priorities include: (i) in vitro validation of the top 15 candidates through cell-based dengue virus inhibition assays encompassing all four serotypes (DENV-1 to DENV-4); (ii) mechanism of action studies employing target deconvolution approaches to identify specific viral proteins engaged by active compounds; (iii) cytotoxicity and selectivity index determination to assess therapeutic windows; (iv) phytochemical characterization of the 174 understudied antiviral plant species to expand the screening library; and (v) investigation of potential synergistic effects between structurally diverse candidates for combination therapy development. Future computational efforts should focus on developing target-specific QSAR models for individual dengue viral proteins (NS3 protease, NS5 polymerase, envelope protein) to enable mechanism-based prioritization and on implementing approaches capable of predicting synergistic interactions in natural product mixtures, as many traditional preparations rely on multi-component formulations.

This work establishes a replicable methodological framework that can be extended to other neglected tropical diseases endemic to biodiversity-rich regions, including Zika virus, chikungunya, and yellow fever. The integration of computational screening, traditional knowledge, ethnopharmacological databases, and biodiversity informatics presented here offers a sustainable model for pharmaceutical bioprospecting that could benefit both global health and local communities.

In conclusion, this pioneering study not only identifies 15 promising anti-dengue candidates from Colombian flora with exceptional combined profiles of predicted potency (pActivity > 6.0), drug-likeness (QED ≥ 0.5), and structural diversity, but also demonstrates how modern computational approaches integrated with comprehensive SAR analysis can systematically unlock the pharmaceutical potential of megadiverse countries. The predominance of structurally novel chemotypes (100% of High-High compounds with Tanimoto < 0.5, comprising 144 novel scaffolds) establishes Colombian biodiversity as a rich source of potentially innovative antiviral mechanisms, with immediate implications for intellectual property development and positioning Colombia as a potential leader in natural product pharmaceutical discovery. As Colombia continues to face the dual challenges of persistent dengue burden (172.9 cases per 100,000 population) and accelerating biodiversity loss, this research provides both practical solutions for drug discovery and compelling economic incentives for conservation. The convergence of machine learning, structure–activity relationship analysis, ethnopharmacological knowledge, and biodiversity informatics presented here establishes a replicable framework for natural product-based drug discovery, with immediate application potential for dengue and extensibility to other neglected tropical diseases affecting biodiversity-rich regions worldwide.

## Figures and Tables

**Table 1:** Performance comparison of different modeling approaches.

Model	MCC	Balanced Acc	ROC-AUC
Ensemble (soft voting)	0.584	0.709	0.901
XGBoost (optimized)	0.583	0.683	0.896
LightGBM (optimized)	0.582	0.708	0.890
ExtraTrees (optimized)	0.580	0.718	0.897
XGBoost (baseline)	0.572	0.681	0.896
ExtraTrees (baseline)	0.559	0.702	0.886
RandomForest (baseline)	0.543	0.682	0.886

**Note:** All optimized models were tuned using Bayesian optimization with 50 trials [29]. The optimized XGBoost model was selected as the final classifier because it exhibited nearly identical predictive performance to the ensemble model while providing lower computational demands, improved reproducibility, and enhanced interpretability. This trade-off between accuracy and model parsimony makes XGBoost particularly suitable for cheminformatics applications, where transparency and scalability are essential.

**Table 2:** Top 15 prioritized candidates from Colombian medicinal flora with dual-endpoint high potency and favorable drug-likeness profiles.

#	Compound Name	Plant Source	Chemical Class	pIC50	pEC50	QED	MW (Da)	LogP	HBA	HBD	TPSA (U)	Novelty	Tanimoto
1	Incartine	*Hippeastrum puniceum*	Alkaloid (Indole)	6.84	6.13	0.83	312.4	2.1	4	2	67.8	Novel	0.28
2	Bilobalide	*Ginkgo biloba*	Terpenoid (Sesquiterpene)	6.78	6.07	0.56	326.3	1.8	6	0	92.5	Novel	0.17
3	Indican	*Indigofera suffruticosa*	Alkaloid (Indole glycoside)	6.73	6.11	0.51	349.3	0.9	7	5	141.2	Novel	0.22
4	Epicolactone	*Euphorbia hirta*	Terpenoid (Diterpene)	6.71	6.94	0.51	348.4	2.4	5	3	86.9	Novel	0.2
5	Byzantionoside B	*Hypericum perforatum*	Terpenoid glycoside	6.65	6.5	0.54	446.5	0.6	9	6	155.1	Novel	0.19
6	3-Acetylnerbowdine	*Lycoris radiata*	Alkaloid (Amaryllidaceae)	6.58	6.82	0.8	329.4	1.7	5	1	70.4	Training-like	0.62
7	Bicyclooctane carboxylic acid derivative	*Schisandra chinensis*	Terpenoid derivative	6.28	6.01	0.53	302.4	2.8	4	2	63.6	Novel	0.25
8	Dihydrofumariline	*Corydalis cava*	Alkaloid (Isoquinoline)	6.19	6.86	0.78	355.4	2.2	6	2	75.3	Training-like	0.58
9	Trifolirhizin	*Sophora flavescens*	Flavonoid glycoside	6.12	6.47	0.52	416.4	0.8	9	6	150.2	Novel	0.24
10	(6R,9R)-3-Oxo-alpha-ionol glucoside	*Rosa damascena*	Terpenoid glycoside	6.06	6.2	0.51	370.4	0.5	7	4	127.4	Novel	0.21
11	Hippeastrine	*Hippeastrum puniceum*	Alkaloid (Amaryllidaceae)	6.05	6.76	0.57	301.3	1.5	5	2	72.5	Training-like	0.65
12	9-Hydroxy-7-megastigmen-3-one glucoside	*Vaccinium myrtillus*	Terpenoid glycoside	6.04	6.68	0.52	372.4	0.7	7	5	134.8	Novel	0.23
13	Lycorine	*Hippeastrum puniceum*	Alkaloid (Amaryllidaceae)	6.02	6.35	0.69	287.3	1.3	4	1	59.7	Training-like	1
14	Digoxigenin	*Digitalis purpurea*	Steroid (Cardiac glycoside aglycone)	5.98	6.42	0.54	374.5	2.5	5	3	86.2	Novel	0.26
15	Pseudolycorine	*Hippeastrum puniceum*	Alkaloid (Amaryllidaceae)	5.95	6.28	0.66	287.3	1.4	4	1	59.7	Training-like	1

## Data Availability

The data presented in this study are openly available.

## Supplementary Materials

The following supporting information can be downloaded at: https://www.mdpi.com/article/10.3390/ph18121906/s1. Table S1: Colombian medicinal plants with antiviral activity, *n* = 358 species; Table S2: Comprehensive model performance metrics with cross-validation results; Table S3: Complete virtual screening results, *n* = 3267 compounds; Table S4: Top priority compounds with detailed properties; Table S5: ADMET properties for the 15 prioritized compounds; Table S6: ADMET scores clean; Table S7: Applicability domain assessment for 15 prioritized compounds including leverage values, Tanimoto similarity, and tier classification; Table S8: Sensitivity analysis of activity thresholds showing class distribution under alternative schemes; Table S9: Hold-out validation metrics including per-class performance; Table S10: Murcko scaffold analysis: 145 unique scaffolds with frequency and novelty assessment; Figure S1: Chemical space visualization and applicability domain; Figure S2: Bayesian optimization and hyperparameter analysis; Figure S3: Optimization convergence analysis: MCC vs. trial number demonstrating 99.6% performance at trial 50; Figure S4: Y-randomization results: distribution of permuted MCC values vs. real model MCC; Figure S5: Learning curves: training and validation MCC as function of training set size; Figure S6: Hold-out validation confusion matrix, *n* = 407; Data S1: Training dataset; Data S2: Colombian phytochemical library; Data S3: Trained models including optimized XGBoost model, feature scaler, variance selector, and model parameters (ZIP format, ~3.2 MB); Code S1: Complete computational pipeline including data preprocessing, QSAR model development, Bayesian optimization, and virtual screening workflow (Python scripts with detailed documentation available at https://github.com/Sergio111999/QSAR_DENV#qsar-denv-anti-dengue-drug-discovery-from-colombian-medicinal-flora).

## Abbreviations

The following abbreviations are used in this manuscript:

AD	Applicability Domain
ChEMBL	Chemical Database of the European Molecular Biology Laboratory
DENV	Dengue Virus
EC50	Half-maximal Effective Concentration
ECFP4	Extended Connectivity Fingerprint (radius 4)
HBA	Hydrogen Bond Acceptors
HBD	Hydrogen Bond Donors
IC50	Half-maximal Inhibitory Concentration
LogP	Logarithm of Partition Coefficient (octanol-water)
MACCS	Molecular ACCess System (fingerprint)
MCC	Matthews Correlation Coefficient
ML	Machine Learning
MW	Molecular Weight
NS2B-NS3	Non-Structural Protein 2B-3 (dengue viral protease)
NS3	Non-Structural Protein 3 (dengue viral protease)
NS5	Non-Structural Protein 5 (dengue viral polymerase)
pActivity	Negative logarithm of activity (−log10[Activity in M])
pEC50	Negative logarithm of EC50 (−log10[EC50 in M])
pIC50	Negative logarithm of IC50 (−log10[IC50 in M])
PC1, PC2	Principal Component 1, 2
PCA	Principal Component Analysis
QED	Quantitative Estimate of Drug-likeness
QSAR	Quantitative Structure–Activity Relationship
RDKit	Open-source cheminformatics toolkit
ROC-AUC	Receiver Operating Characteristic—Area Under the Curve
RotBonds	Rotatable Bonds
SAR	Structure–Activity Relationship
SMILES	Simplified Molecular Input Line Entry System
Tanimoto	Tanimoto Similarity Coefficient
TPSA	Topological Polar Surface Area
UMAP	Uniform Manifold Approximation and Projection
XGBoost	eXtreme Gradient Boosting
