# Supplementary figures and images for: Machine Learning-Based QSAR Screening of Colombian Medicinal Flora for Potential Antiviral Compounds Against Dengue Virus: An In Silico Drug Discovery Approach
Source: Pharmaceuticals (Basel). 2025 Dec 18;18(12):1906. doi: 10.3390/ph18121906 (PMC12736152; doi:10.3390/ph18121906)

**B) Chemical Space (ECFP4)**  
**Novel ★ vs Training-like ●**

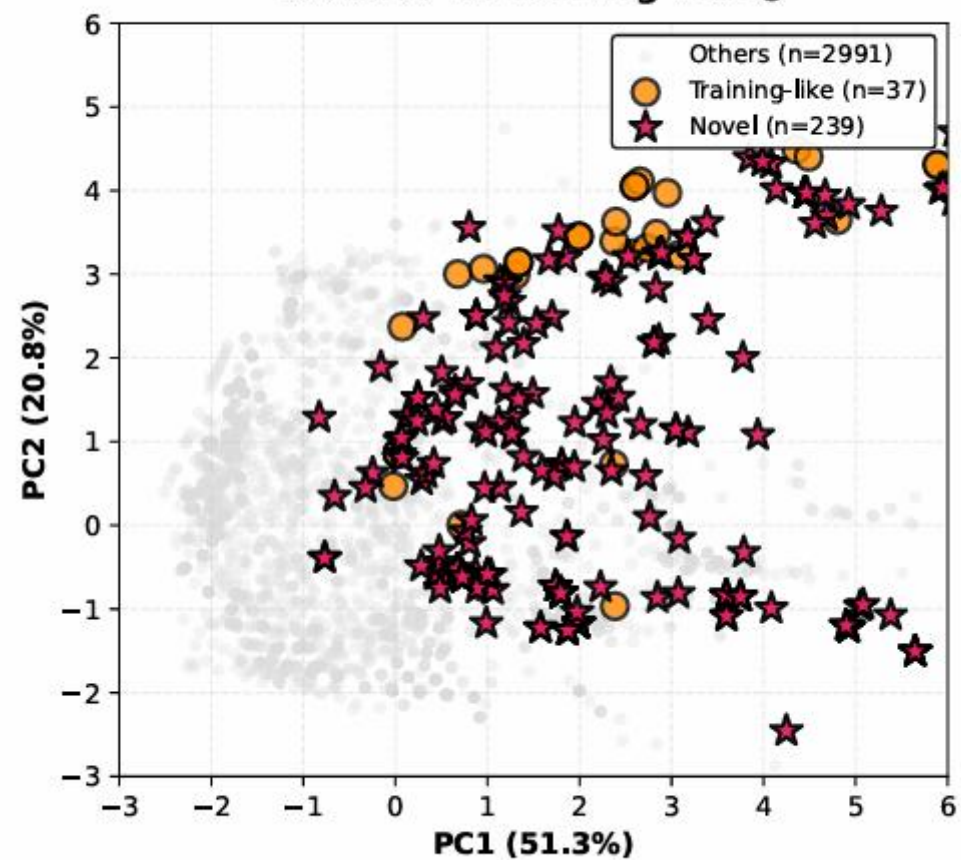

Supplement: Supplementary file 1 [file pharmaceuticals-18-01906-s001.zip › Figure S1 Chemical space visualization and applicability domain.pdf]

# Comprehensive Model Optimization and Validation Analysis

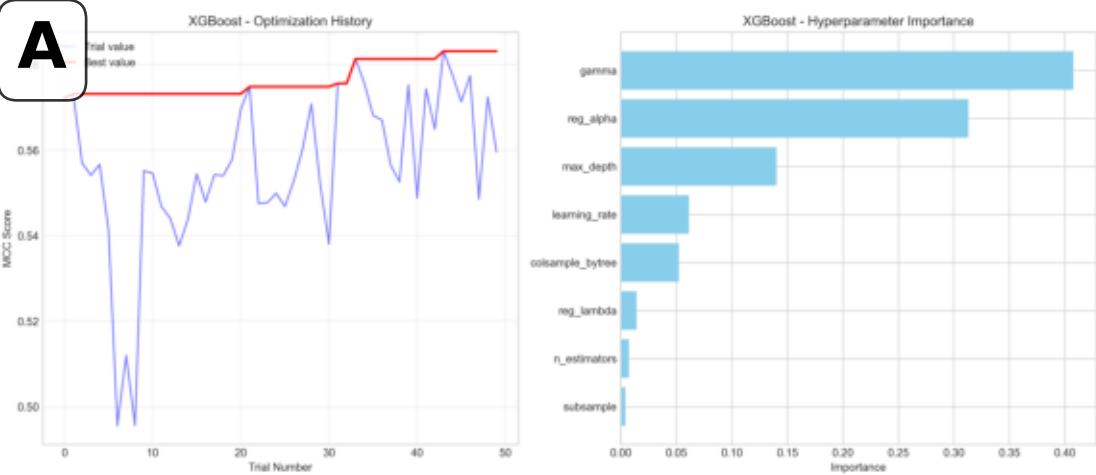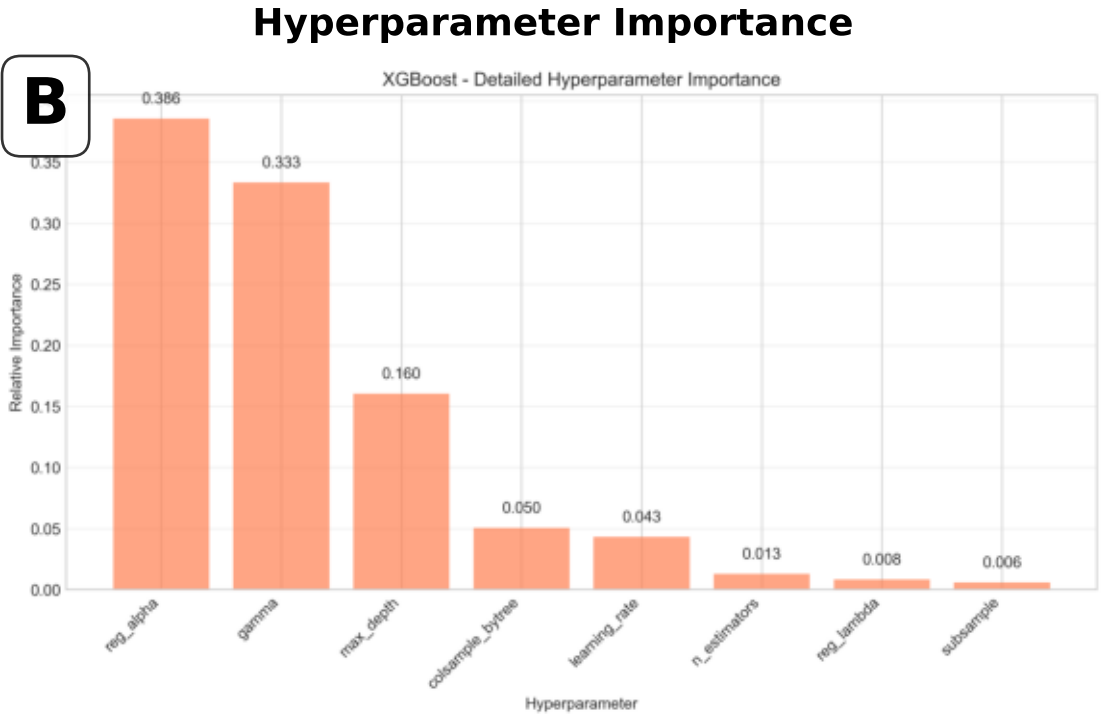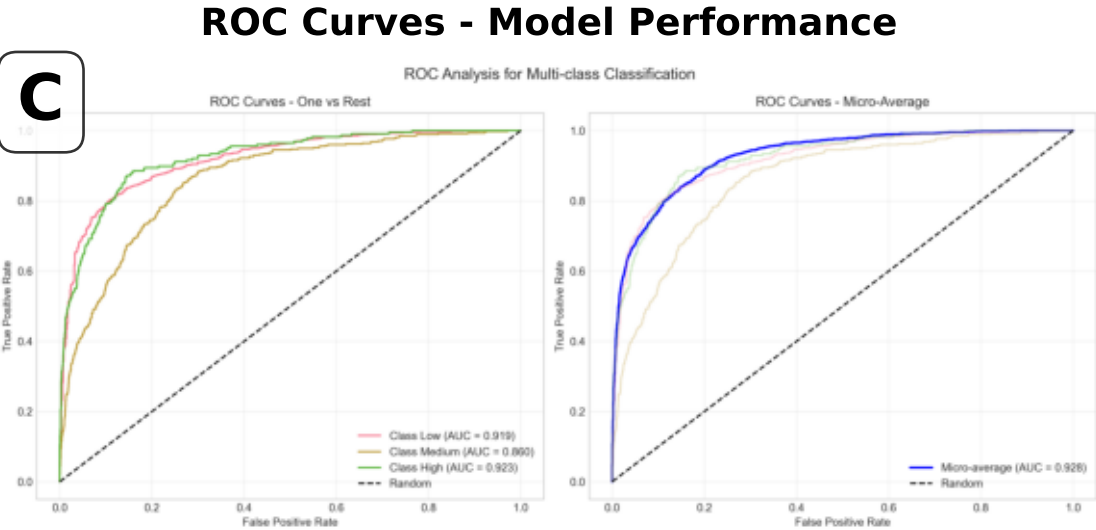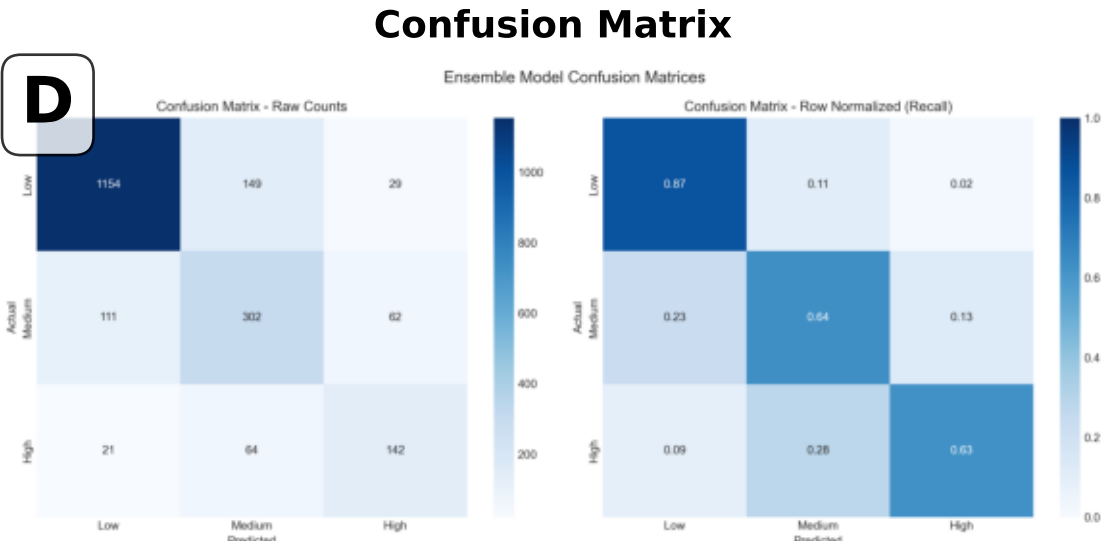

Supplement: Supplementary file 1 [file pharmaceuticals-18-01906-s001.zip › Figure S2 Bayesian optimization and hyperparameter analysis.pdf]

# Análisis de Convergencia de Optimización

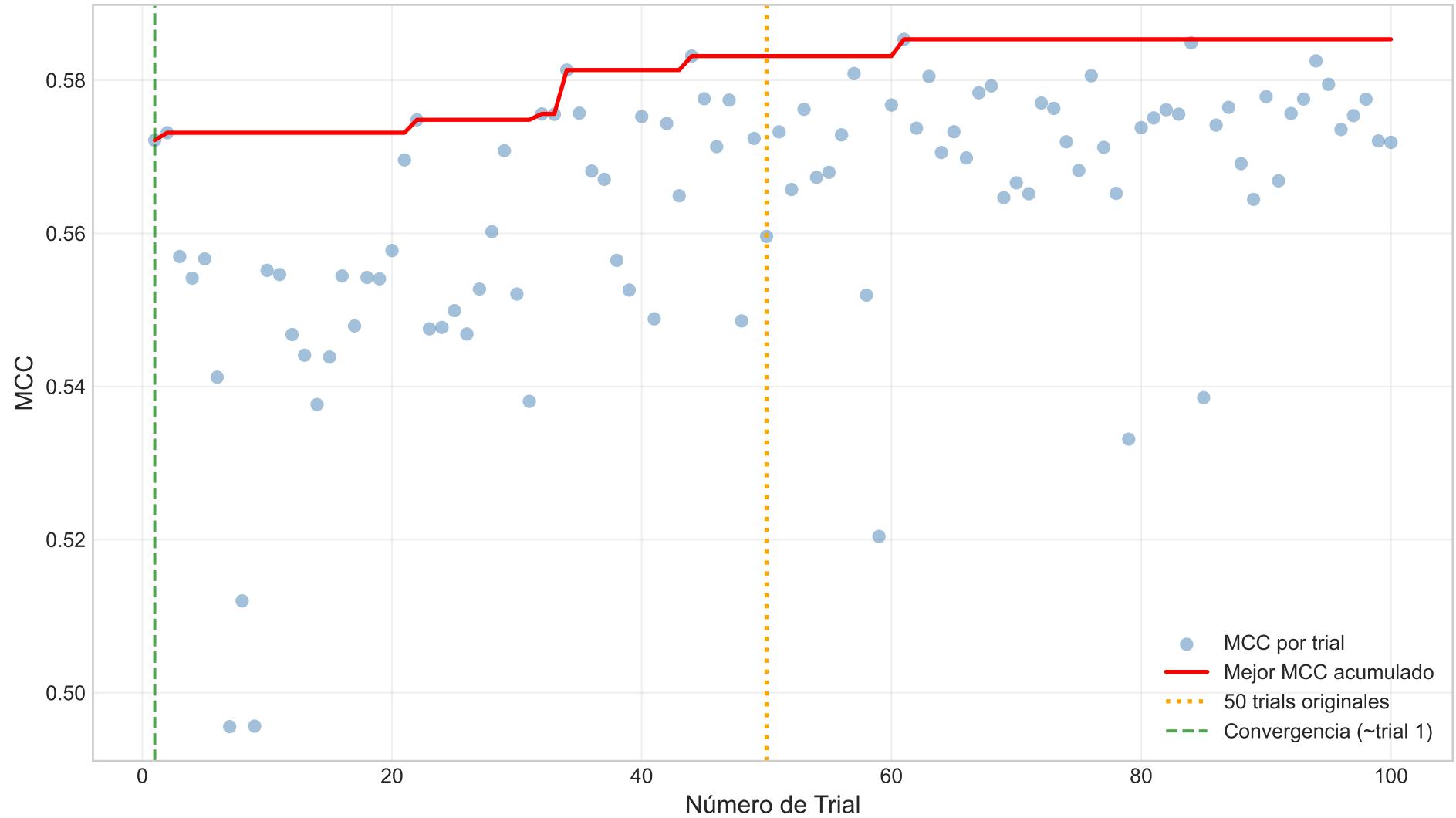

Supplement: Supplementary file 1 [file pharmaceuticals-18-01906-s001.zip › Figure S3 Optimization convergence analysis MCC vs. trial number demonstrating 99.6% per-formance at trial 50.pdf]

# Test de Y-Randomization ( $p < 0.0099$ )

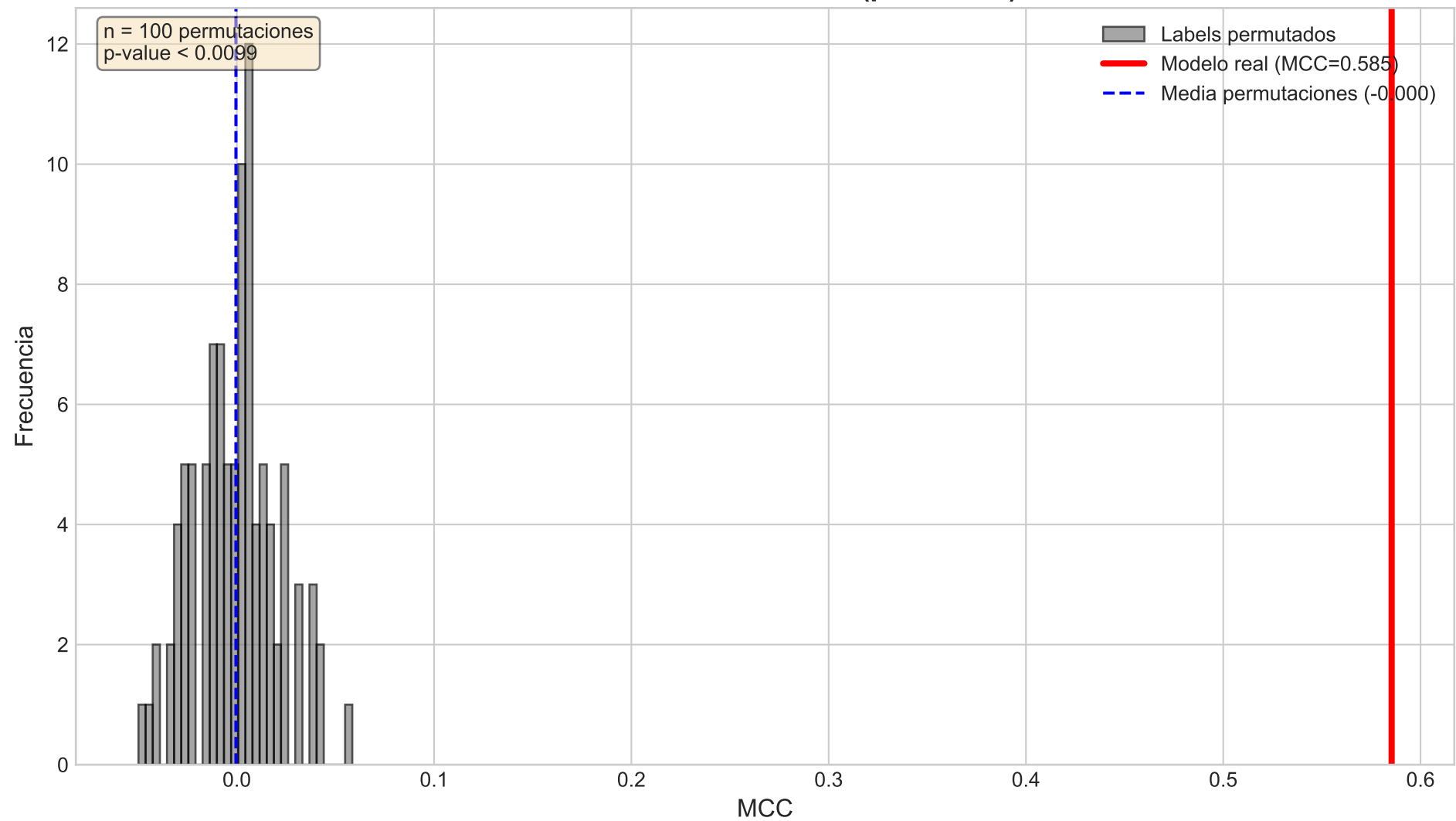

Supplement: Supplementary file 1 [file pharmaceuticals-18-01906-s001.zip › Figure S4 Y-randomization results distribution of permuted MCC values vs. real model MCC.pdf]

## Learning Curves

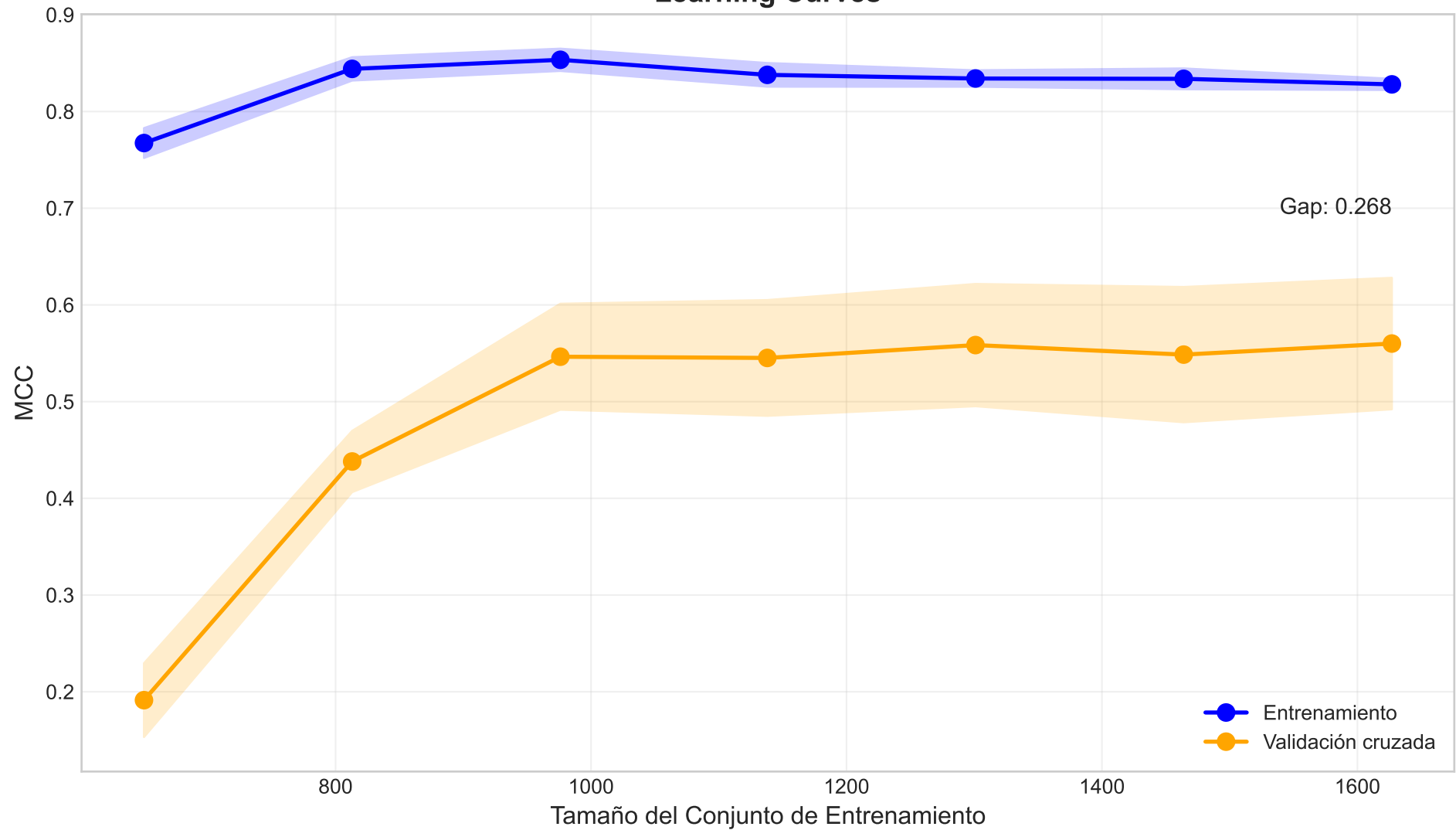

Supplement: Supplementary file 1 [file pharmaceuticals-18-01906-s001.zip › Figure S5 Learning curves training and validation MCC as function of training set size.pdf]

**Matriz de Confusión Hold-out**  
**MCC = 0.576**

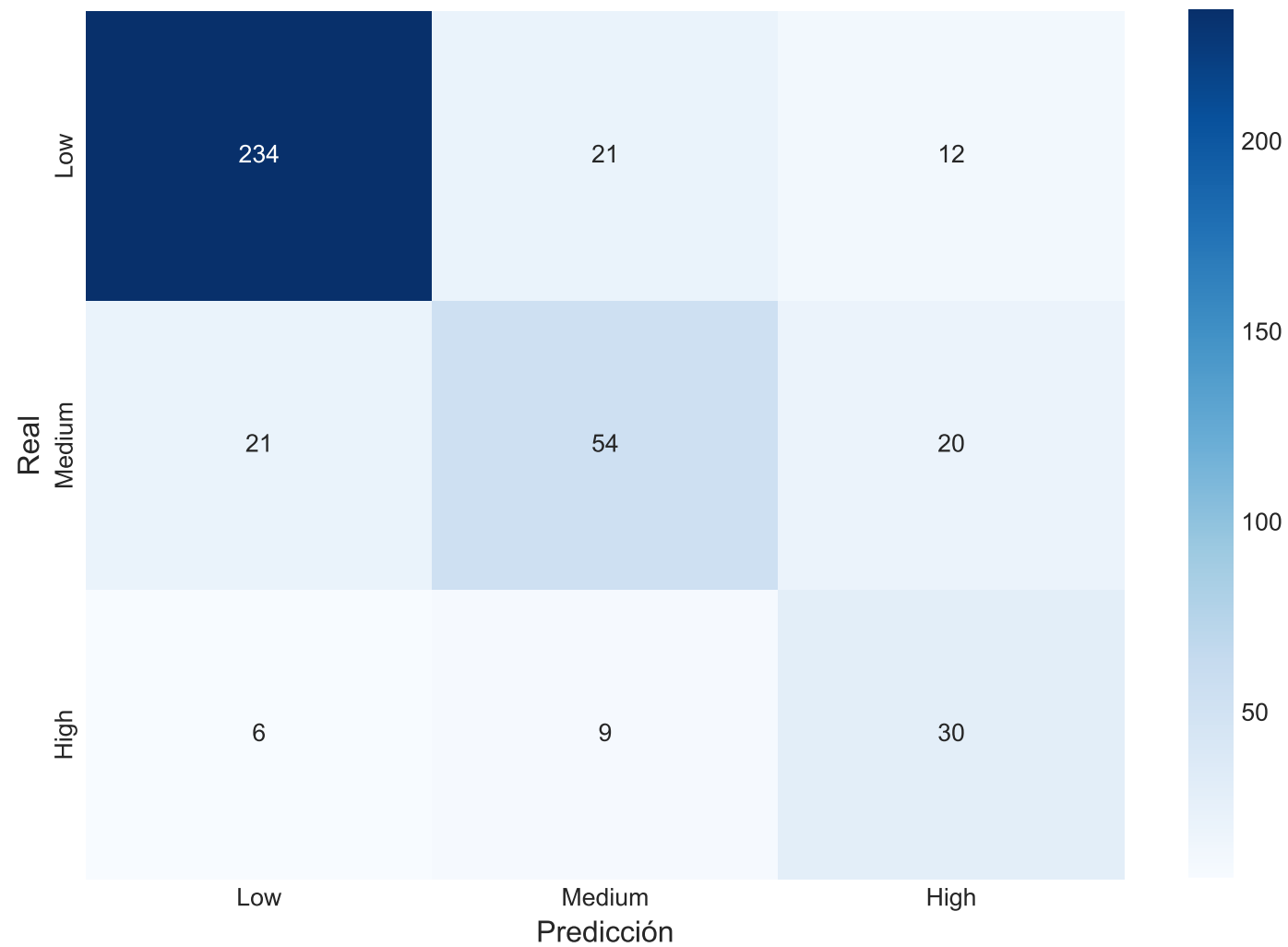

Supplement: Supplementary file 1 [file pharmaceuticals-18-01906-s001.zip › Figure S6 Hold-out validation confusion matrix.pdf]
